# The Role of Macronutrients, Micronutrients and Flavonoid Polyphenols in the Prevention and Treatment of Osteoporosis

**DOI:** 10.3390/nu14030523

**Published:** 2022-01-25

**Authors:** Monika Martiniakova, Martina Babikova, Vladimira Mondockova, Jana Blahova, Veronika Kovacova, Radoslav Omelka

**Affiliations:** 1 Department of Zoology and Anthropology, Faculty of Natural Sciences and Informatics, Constantine the Philosopher University in Nitra, Tr. A. Hlinku 1, 949 01 Nitra, Slovakia; mmartiniakova@ukf.sk (M.M.); vkovacova@ukf.sk (V.K.); 2 Department of Botany and Genetics, Faculty of Natural Sciences and Informatics, Constantine the Philosopher University in Nitra, Tr. A. Hlinku 1, 949 01 Nitra, Slovakia; martina.babikova@ukf.sk (M.B.); vmondockova@ukf.sk (V.M.); jana.blahova@ukf.sk (J.B.)

**Keywords:** osteoporosis, nutrition, macronutrients, micronutrients, flavonoid polyphenols, prevention, treatment

## Abstract

Osteoporosis is considered an age-related disorder of the skeletal system, characterized primarily by decreased bone mineral density (BMD), microstructural quality and an elevated risk of fragility fractures. This silent disease is increasingly becoming a global epidemic due to an aging population and longer life expectancy. It is known that nutrition and physical activity play an important role in skeletal health, both in achieving the highest BMD and in maintaining bone health. In this review, the role of macronutrients (proteins, lipids, carbohydrates), micronutrients (minerals—calcium, phosphorus, magnesium, as well as vitamins—D, C, K) and flavonoid polyphenols (quercetin, rutin, luteolin, kaempferol, naringin) which appear to be essential for the prevention and treatment of osteoporosis, are characterized. Moreover, the importance of various naturally available nutrients, whether in the diet or in food supplements, is emphasized. In addition to pharmacotherapy, the basis of osteoporosis prevention is a healthy diet rich mainly in fruits, vegetables, seafood and fish oil supplements, specific dairy products, containing a sufficient amount of all aforementioned nutritional substances along with regular physical activity. The effect of diet alone in this context may depend on an individual’s genotype, gene-diet interactions or the composition and function of the gut microbiota.

## 1. Introduction

Osteoporosis, a common age-related skeletal disorder, is represented by reduced bone mineral density (BMD), deterioration of bone microarchitecture with an increased risk of fragility fractures [1]. Such fractures, located predominantly in the wrist, hip and spine, are associated with considerable pain and suffering, disability and sometimes even death [2]. Osteoporosis, often referred to a silent epidemic, affects millions of people around the world [3]. By 2050, more than 30 million people in Europe will be affected by osteoporosis [4] due to an aging population and longer life expectancy. Although the entire population is at risk, the likelihood of developing osteoporosis increases depending on a variety of factors, including gender, age, family history or fracture, and Caucasian or Asian ethnicity [5].

It has been estimated that 50–85% of the variation in BMD at middle-age is genetically determined. However, the heritability of both bone loss and fractures decreases with increasing age and lifestyle factors becoming more important [6]. It is difficult to determine how much of a lifestyle effect is affected by diet alone, due to difficulties in quantifying environmental exposures, both current and lifelong. In addition, the impact of diet on bone health may be more complex and it may depend on the genotype of the individual, gene-diet interactions or the composition and function of the gut microbiota [7,8].

Age is one of the main risk factors for primary type 1 (postmenopausal) osteoporosis [9]. Primary type 2 (senile) osteoporosis occurs after the age of 75 years and is determined in both women and men at a ratio 2:1 [10]. The mechanism underlying age-related changes in bone quantity and quality occurs at the cellular, molecular and genetic levels [11]. Bone metabolism is altered due to differences in the number, activity and response of bone cells which leads to bone loss and bone fragility. In addition, bone fat marrow accumulation [12], changes in bone regulation, increased cell apoptosis, accumulation of cellular senescence, DNA damages and genomic instability, telomere dysfunction and epigenetic alterations significantly contribute to bone aging [13,14].

**Figure 1:**
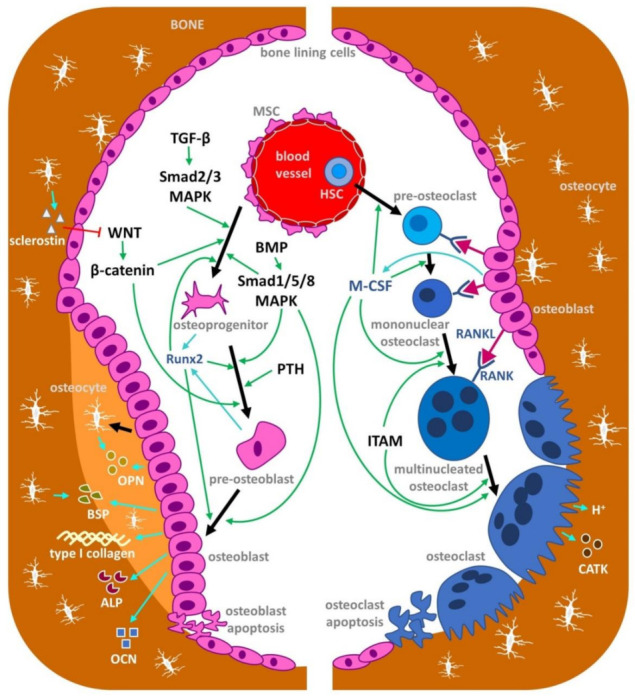
Schematic representation of osteoblast (left) and osteoclast (right) differentiation with the involvement of selected important signaling pathways and regulators. Positive effects are depicted by green arrowheads. The blue arrows and font show the production of selected molecules important in the signaling pathway and also for the processes of bone formation and resorption. Osteoblastogenesis is regulated by different signaling pathways (including TGF-β/BMP, WNT), resulting in activation of downstream transcription factors such as Runx2 and leading to the expression of osteoblastogenic markers by osteoblasts, e.g., type I collagen, ALP, OPN, BSP and OCN. The canonical WNT pathway activates downstream signaling cascades, resulting in β-catenin translocation into the nucleus which enhances osteoblastogenic target gene expression. TGF-β and BMPs are secreted growth factors belonging to the TGF-β superfamily, which play an essential role in development, tissue homeostasis and regeneration. Canonical TGF-β signaling mobilizes transcription factors Smad2 and Smad3 to interact with other transcription factors and induces Runx2-mediated gene expression. Similar to TGF-β, canonical BMP signaling also transmits signals through Smad transcription factors. Moreover, BMPs and TGF-β can induce Runx2 through the MAPK signaling pathway. Osteocytes represent a differentiated stage of the osteoblast lineage and they have key regulatory roles in bone and mineral homeostasis. Osteoclasts differentiate from cells of the monocyte/macrophage lineage in response to the osteoclastogenic cytokines M-CSF, RANKL and ITAM. As a result, mononuclear osteoclast precursors finally fuse into mature polykaryons which produce protons and proteolytic enzymes (especially cathepsin K) to dissolve bone minerals and degrade bone matrix proteins. MSC, mesenchymal stem cell; TGF-β, transforming growth factor beta; BMP, bone morphogenetic protein; MAPK, mitogen-activated protein kinase; Runx2, runt-related transcription factor 2; WNT, Wnt glycoproteins; PTH, parathyroid hormone; OPN, osteopontin; BSP, bone sialoprotein; ALP, alkaline phosphatase; OCN, osteocalcin; HSC, hematopoietic stem cell; M-CSF, macrophage-colony stimulating factor; ITAM, immunoreceptor tyrosine-based activation motif; RANK, receptor activator of nuclear factor κΒ; RANKL, receptor activator of nuclear factor κΒ ligand; CATK, cathepsin K.

The mechanisms associated with bone loss are well known, including the role of pro-inflammatory cytokines, e.g., tumor necrosis factor α (TNFα), interleukin-1 (IL-1), interleukin-6 (IL-6), in activating bone resorption by osteoclasts (bone-resorbing cells) and inhibiting bone formation by osteoblasts (bone-forming cells) [15]. Bone formation occurs mainly on periosteal surfaces, whereas bone resorption is usually present at endosteal surfaces. Osteoclast activation and differentiation is regulated by a variety of molecular signals, one of the most studied being the receptor activator for nuclear factor-kappa B (RANK) ligand (RANKL). RANKL is produced by osteocytes as well as osteoblasts and stimulates RANK on osteoclast progenitors [16]. Furthermore, estrogen loss during menopause can be consistent with an elevated expression of RANKL and reduced expression of osteoprotegerin (OPG), its natural antagonist by osteoblasts and osteocytes, thereby raising bone resorption in both trabecular and cortical bone compartments [10,17]. The role of bone formation is equally important in the process of bone fragility and is controlled by sclerostin, which is expressed by osteocytes and acts as an inhibitor of the Wnt/β-catenin signaling pathway, a strong stimulus for osteoblast differentiation [15]. Schematic representation of osteoblast and osteoclast differentiation with the involvement of selected important signaling pathways and regulators is illustrated in Figure 1.

In addition to pharmacotherapy, nutritional supplements and physical exercise are commonly used to prevent and treat osteoporosis [3,18]. Pharmacological treatment includes anti-resorptive drugs (e.g., bisphosphonates, estrogen replacement therapy, selective estrogen receptor modulators, calcitonin, denosumab, calcium and vitamin D supplementation), and anabolic drugs (e.g., teriparatide, abaloparatide, strontium ranelate, romosozumab) [19,20]. Nutrition plays a dominant role in skeletal health, both in achieving the highest BMD and in maintaining bone health [21]. Therefore, a balanced diet and good nutritional program can also prevent osteoporosis. The intake of macronutrients, vitamins and minerals is often below the recommended values, especially if the disease is active [22]. Calcium (Ca) and vitamin D are the most frequently discussed nutrients with respect to BMD. In addition, vitamins (e.g., C, K), minerals (e.g., Ca, phosphorus (P), magnesium (Mg)), flavonoid polyphenols (e.g., quercetin, rutin, luteolin, kaempferol, naringin) are also involved in bone formation. Exercises to tone deambulatory muscles and exercises to improve proprioception can help prevent falls. Moreover, physical exercise can stop or reverse osteoporosis due to its anabolic effects [10]. This review characterizes the role of essential macronutrients, micronutrients and flavonoid polyphenols in relation to osteoporosis prevention and treatment. Moreover, the importance of different naturally available nutrients, whether in the diet or in food supplements is highlighted.

## 2. Macronutrients and Osteoporosis

Diets consist mainly of macronutrients—proteins, lipids and carbohydrates. In many cases, the way that these macronutrients influence bone health reflects their ability to change Ca metabolism and skeletal homeostasis [23]. Table 1 shows the amounts of macronutrients and micronutrients in different types of foods.

### 2.1. Proteins

Proteins are complex molecules that have a variety of functions in the body. They can be beneficial as well as harmful to bone health depending on the amount of protein ingested (low-protein diet vs. high-protein diet) and the source of protein (plant vs. animal) [32]. Dietary protein is a key nutrient for skeletal health because it influences bone in several ways: (a) it forms a large component of organic bone matrix, (b) regulates serum levels of insulin-like growth factor 1 (IGF-1) and (c) may affect Ca metabolism [23]. In addition, proteins are important components of bone, accounting for approximately 30% of bone mass, and 50% of bone volume. They can also influence bone metabolism since it depends on dietary protein intake [33].

The current recommended dietary allowance (RDA) for protein is 0.8 g/kg body weight (bw)/day for adults (1.5 g/kg bw/day for children, 1.0 g/kg bw/day for adolescents and older people) [34,35]. According to Wallace and Frankenfeld [36], protein supplementation above the current RDA may play a positive role in preventing osteoporotic fractures and bone loss. Observational studies clearly demonstrate that higher dietary protein consumption is associated with higher BMD in middle-aged and older adults [37,38,39] and may have a protective impact against vertebral and femoral bone loss [40]. Similarly, elevated protein intake (approximately 0.8–1.3 g/kg bw/day) had no detrimental effect on bone quality in healthy adults as well [41].

Typical proteins of animal origin are present in meat, fish, poultry, eggs and dairy products. They are often called “complete” proteins for their sufficient amount of essential amino acids [42]. Vegetable proteins can be obtained from plant sources, such as legumes, tofu, soy, tempeh, seitan, nuts and seeds. They may have poorer nutritional quality due to their variable amino acid profile, e.g., lower proportion of lysine, cysteine or methionine [43,44]. Various in vitro studies suggest that amino acids can affect bone health by a variety of mechanisms. Osteoblast growth and differentiation is partially supported via a stimulation of insulin secretion by alanine, lysine, arginine, leucine and glutamine [45,46,47]. Lysine and arginine have shown a positive outcome for nitric oxide (NO) production and type I collagen synthesis [48] with the potential to be used in osteoporosis prevention.

Different types of studies attempted to find preferred sources of protein for nutritional interventions, but without clear conclusions. On the one hand, Iguacel et al. [49] determined lower BMD in the femoral neck and lumbar spine of vegetarians and vegans compared to omnivores, and on the other hand, no significant differences between animal and plant protein consumption were observed in relation to BMD, bone mineral content (BMC), bone turnover markers and hip fractures [50,51]. In any case, it is clear that the impact of proteins on bone health may vary according to Ca intake. Higher protein intake increased BMD and protected against the risk of fragility fractures in adults with sufficient Ca intake [51].

Insufficient dietary protein intake can lead to muscle wasting, resulting in unintentional loss of body weight due to accelerated muscle protein degradation and reduced protein synthesis. It is a clinically significant complication of many chronic diseases, including osteoporosis [52]. Lean muscle mass affects not only overall BMD but also key cross-sectional bone parameters related to bone strength. Therefore, bone loss and fragility fractures in older individuals are often preceded by loss of muscle mass and strength [53]. Several epidemiological studies have supported the close association between low muscle mass and osteoporosis. Verschueren et al. [54] reported that middle-aged and elderly men with sarcopenia (a disease characterized by progressive and general loss of muscle mass with either low muscle strength or physical performance) had significantly lower BMD and were more likely to develop osteoporosis compared with those without sarcopenia. Another study involving both men and women over 65 years showed that low muscle mass was significantly associated with a higher risk of osteoporosis, even after adjusting for potential risk factors [55].

In recent decades, attention has also focused on bioactive peptides, specific fragments arising from proteins during gastrointestinal digestion by enzymatic proteolysis [56,57]. Bioactive peptides are able to directly influence bone regulation by activating signaling pathways and modifying osteoblast functions [58,59]. Administration of collagen peptides in postmenopausal women with reduced BMD was consistent with BMD improvement [60]. Some dairy components, for example, milk casein-derived peptide, also possess osteoprotective activity by reducing RANKL, IL-6 and TNF-α expression [61]. Seafood bioactive peptides, which can act as stimulators and inhibitors of bone formation and resorption, respectively, are considered another source for the prevention of osteoporosis due to immunomodulatory, antioxidant, antihypertensive, osteoprotective and antimicrobial properties [56,62].

### 2.2. Lipids

Lipids are essential macronutrients that have key functions in the body, such as structural units of cell membranes, energy storage, precursors of metabolic compounds involved in inflammatory and immune responses [63]. They are also important for the absorption of fat-soluble vitamins (A, D, E, K). For this reason, the WHO recommends that fats account for 20–35% of total energy intake [64].

According to the number of chemical double bonds, fatty acids can be divided into saturated fatty acids (SFAs), monounsaturated fatty acids (MUFAs) and polyunsaturated fatty acids (PUFAs) [64,65]. Common SFAs are myristic, palmitic and stearic acids, which affect cholesterol metabolism and are found in foods rich in animal fats (e.g., meat, dairy products) or in vegetable fats (e.g., palm oil, coconut oil) [66]. SFAs have been widely considered to be harmful to health, especially in cardiovascular disorders. The most common MUFA is oleic acid, present mainly in olive oil and meat (e.g., beef, pork) [67]. MUFA-rich diet may have beneficial health effects, especially in the presence of coronary heart disease and type 2 diabetes mellitus [68,69]. The results by Schwingshackl and Hoffmann [70] indicate an overall risk reduction of all-cause mortality (11%), cardiovascular mortality (12%), cardiovascular events (9%) and stroke (17%) due to olive oil consumption. However, MUFAs of mixed animal and plant sources did not have any significant effect on aforementioned parameters. These data provide evidence that the source and origin of MUFA in a specific diet should be considered in order to assess the potential benefits of this type of fatty acid. Among PUFAs, α-linolenic acid (*n*-3) and linoleic acid (*n*-6) are the most important. The human body cannot synthesize them and therefore they must be obtained from foods. The main sources of these acids are vegetable oils, such as sunflower oil, rapeseed oil and soybean oil. Functionally important fatty acids, such as arachidonic acid (*n*-6), eicosapentaenoic acid (*n*-3) and docosahexaenoic acid (*n*-3), can be synthesized from linoleic acid and α-linolenic acid, although not very efficiently [64]. Some PUFAs can have a beneficial role as biological mediators associated with cardiovascular diseases [71].

Dietary lipids can also influence bone health. Increased lipid intake may result in decreased BMD and an elevated risk of fractures [72,73]. A negative relationship between SFAs consumption and femoral neck BMD has been determined in men over 50 years of age [74]. In postmenopausal women, higher SFAs intake was associated with elevated hip fracture risk [75]. Garcia-Martinez et al. [76] revealed that intake of MUFAs, which are derived from olive oil in the Mediterranean-style diet, is associated with increased BMD at the distal end of the radius in both sexes. According to Roncero-Martin et al. [77], dietary olive oil administration was positively associated with lumbar spine BMD in adult women (23–81 years). The beneficial effects of olive oil on BMD have been attributed to the high contents of vitamin E and phenolic compounds. Although total PUFA intakes have been positively associated with bone health, complex interactions between individual fatty acid and bone are gaining attention based on studies published from the Framingham cohorts. In summary, no significant correlations were recorded with consumption of individual PUFA and BMD in both sexes [78]; however, women who simultaneously received eicosapentaenoic and docosahexaenoic acids had enhanced femoral neck BMD and elevated intake of arachidonic acid. This interaction was also determined in men where the individuals in the highest quartile of arachidonic acid intakes lost more hip BMD than those with the lowest intakes, but only among individuals with low eicosapentaenoic and docosahexaenoic acids consumption [79]. Therefore, the protective effects of an arachidonic acid-rich diet may depend on adequate intake of eicosapentaenoic and docosahexaenoic acids. According to Tartibian et al. [80], *n*-3 fatty acids supplementation in combination with aerobic exercise increased BMD in postmenopausal women. Dietary α-linolenic acid had a protective role against hip fractures in older adults [81]. Men in the highest income quartile had an 80% lower risk of hip fracture compared to those in the lowest income quartile. Both men and women with the highest plasma concentrations of α-linolenic acid showed a 51% lower risk of fractures. Some seafood and fish oil supplements are high in PUFA (especially the *n*-3 group of fatty acids), so it is recommended that they should be included in the diet in middle age to ensure better BMD. Recent findings show that higher intake of fish is associated with a higher BMD or lower risk of fragility fractures in women [82]. The supplementation with 4% highly purified concentrated fish oil was effective in maintaining BMD during aging [83].

There are many mechanisms by which lipids can exert their effects. One of them is hyperinsulinemia, which may be consistent with hypercalciuria, high urinary Mg levels and a negative balance of Ca and Mg [21]. Other mechanisms include reduced Ca absorption and elevated retinol intake, which can cause an increased bone resorption. High fat diet also decreases bone formation and mineral apposition, enhances sclerostin expression and damages osteocyte canaliculi network [84]. An increased intake of lipids can also be associated with a diet low in other important nutrients, which can also affect bone health [85].

The interaction between obesity and osteoporosis is not fully understood yet. The association between obesity and fracture risk may be skeletal site- and sex-specific, but results among studies are inconsistent [86]. While several researches report increased BMD in obese patients, it appears that altered bone quality may be a major determinant of fracture risk in this population [87]. This is due to a combination of several factors including increased marrow adipogenesis at the expense of osteoblastogenesis, pro-inflammatory cytokine activity, excessive leptin secretion and reduced adiponectin [88]. Although weight reduction is recommended to reduce obesity-related comorbidities, it may also induce bone loss and increases risk of fragility fractures, especially those of the ankle, upper leg and humerus [89]. Management strategies to attenuate bone loss during weight reduction include physical exercise and dietary interventions with Ca, vitamin D and protein intake [90]. Consumption of phytoestrogens and functional foods (e.g., dried plum, flaxseeds, garlic) can also be beneficial in this context [91].

### 2.3. Carbohydrates

Dietary carbohydrates are macronutrients with a range of physical and physiological properties and health benefits. They are found in fruits, grains, vegetables and milk products. These compounds are present in foods as low molecular weight mono- and disaccharides, intermediate molecular weight oligosaccharides and high molecular weight polysaccharides [92]. Dietary fibers, a complex group of carbohydrates and lignin, also have a significant effect on health. Fiber is not hydrolyzed by human enzymes and is therefore not digested or absorbed in the human body. However, while insoluble fiber (such as cellulose and hemicellulose) passes intact through the digestive tract, soluble fiber can be fermented by gut bacteria [93].

Carbohydrates supply energy to host cells and the intestinal microbiome. Fermentable carbohydrates (especially mono- and disaccharides) and fiber can play an important role in osteoporosis prevention [22]. According to Cohen et al. [94], high carbohydrate intake was consistent with a reduced BMD at the distal end of the radius; however, the correlation was significant only for related monosaccharides and disaccharides. On the other hand, Kato et al. [72] revealed a positive association between total carbohydrate intake and osteoporotic fractures in postmenopausal women.

In diets high in refined (processed) sugar, the monosaccharide glucose and disaccharide sucrose are among those that have been thoroughly investigated. Current evidence suggests that they could influence bone growth and strength. Lower tibial and femoral bone strength was diagnosed in sucrose-fed rats [95]. Glucose at high concentrations adversely affected osteoblast proliferation and differentiation in vitro [96]. In addition, excessive urinary Ca loss was recorded in young adults administered an oral glucose solution [97], which points to the fact that glucose affects Ca metabolism [23]. High-fructose diet also reduced Ca ion transport in animals [98].

Carbonated and sugar-sweetened beverages have been a major source of carbohydrates for humans in recent decades. Consumption of these beverages is often associated with decreased BMD [99]. Some animal studies suggest that changes in bone quality may be due to a concomitant reduction in the consumption of milk and other nutrient-rich fluids [99,100]. In the study by Vartanian et al. [101], consumption of carbonated beverages was negatively related to Ca intake. Tsanzi et al. [99] compared the impact of different sugar-sweetened beverages on bone health of growing rats. According to their results, glucose intake had a more detrimental effect on BMD, BMC and Ca retention versus fructose administration.

It should be noted that not all carbohydrates have a harmful effect on bone quality. Most studies examining the relationship between carbohydrate consumption and BMD have focused on fiber intake, because fiber can prevent a decrease in BMD [21]. It is known that water-soluble fiber administration caused elevated Ca retention in the bone [102]. Many fruits and vegetables contain indigestible carbohydrates, such as inulin-type fructans. A large increase in Ca absorption was observed in young adults (58% increase) as well as in postmenopausal women (42% increase) after inulin supplementation [103]. Moreover, Abrams et al. [104] determined an enhanced BMD and BMC in young adolescent after 1 year of inulin administration.

Carbohydrates among all macronutrients have the greatest impact on postprandial blood glucose levels and therefore monitoring of carbohydrate intake is considered an important strategy in the management of diabetes mellitus [105]. Patients with diabetes mellitus are diagnosed with an increased risk of bone fragility and fractures, similar to osteoporosis. It follows that bone integrity can also be negatively affected by hyperglycemia and diabetic bone disease is considered a significant secondary complication of diabetes [106,107]. It is therefore surprising that bee products have the ability to alleviate diabetic complications, including the improvement of damaged bone structure. Martiniakova et al. [108] reported a protective effect of bee bread against hyperglycemia and diabetic bone disease in Zucker diabetic fatty rats. In addition, honey can also improve glycemic control and reduce diabetic complications [109] due to a wide range of proteins, bioactive peptides, fatty acids, organic acids, phenolic acids, prebiotics, probiotics, fiber, minerals, vitamins, flavonoids and carotenoids.

## 3. Micronutrients and Osteoporosis

Micronutrients include minerals and vitamins that are important for healthy development of the skeletal system, disease prevention and well-being. With the exception of vitamin D, they are not produced in the body and must therefore be taken by food [110]. The most important and studied micronutrients in the prevention and treatment of osteoporosis are Ca and vitamin D. However, other minerals and vitamins (e.g., P, Mg, zinc, selenium, copper, vitamins C, K, A, B) are also involved in bone formation. In our review, from the group of minerals, Ca, P and Mg are characterized, and also vitamins D, C and K are described in more detail. The amount of these micronutrients in different types of foods is shown in Table 1.

### 3.1. Minerals

#### 3.1.1. Calcium

Calcium is the most important nutrient not only for bone health, but it is also essential for neuromuscular activity, heart rate regulation, immune function and other key physiological processes. In the human body, more than 99% of Ca is stored in the bones, specifically in the form of hydroxyapatite crystals [18,23]. Adequate Ca intake is essential for normal skeletal growth and development, as well as for bone mineralization. Conversely, insufficient Ca supplementation is associated with hormonal disorders, leading to age-related bone loss and increased risk of osteoporosis [22,111]. Numerous studies have reported that adequate Ca consumption at different ages is consistent with raised BMD and reduced risk of fractures [22,112,113]. However, individual Ca supplementation to eliminate fracture risk is recommended only in individuals at high risk of either insufficient Ca intake, Ca absorption, or both, in order to achieve the expected benefits to the skeleton, taking into account its potential negative effects, such as an increased risk of kidney stones and myocardial infarction [114,115,116]. This fact was supported by the meta-analysis of Tai et al. [117] who found that Ca supplements alone only slightly enhanced BMD (by 0.7–1.8%) in clinical trials.

Milk and other dairy products are the main source of Ca in the diet, but significant amounts are also found in foods such as salmon, almonds, leafy green vegetables, legumes, tofu, seafood and Ca-fortified foods, especially orange juice [21,118]. Another source of Ca in the diet can be mineral waters enriched with Ca [119]. Heaney’s [120] study revealed that high-Ca mineral waters have the same or slightly better absorbency as milk Ca and should provide useful amounts of bioavailable Ca. The recommended Ca intake varies depending on the age of the individual [121] and acquires values of 1200 mg/day for young adults, 1000 mg/day for women aged 25 to 50 years and 1500 mg/day for postmenopausal women [23,118].

Selected foods with low levels of oxalic acid (e.g., bananas, blueberries, apples, broccoli, cabbage, white rice, eggs, meat, fish, yogurt, cheese, milk, fruit juice) and phytic acid (food processed by several pretreatment methods, such as fermentation, soaking, germination and enzymatic treatment) can contribute to enhanced Ca incorporation into the skeleton. Oxalic acid and phytic acid interfere with the absorption of Ca and the food source containing them is considered a weak source of Ca [21]. An excessive sodium (Na) consumption may also increase urinary excretion of Ca, as Ca and Na compete for reabsorption in the renal tubules [23]. On the contrary, P and vitamin D are effective in increasing Ca intake. The Ca:P ratio is important for proper bone formation. It is common practice to have a Ca:P molar ratio of 1–2:1 [122]. The benefits of simultaneous Ca and vitamin D supplementation in preventing bone loss, reducing bone turnover and non-vertebral fractures are obvious in postmenopausal women [112]. Block et al. [123] demonstrated that a high protein diet (HPD; 45 g protein per meal) causes an increase in urinary Ca and a decrease in the renal reabsorption of Ca. In contrast, sulfur amino acid supplement to a low protein diet (LPD; 15 g protein per meal), in an amount equivalent to those in the HPD, had no effect on Ca excretion or reabsorption within 4 h after meal ingestion. In other study including 24-h urine collections, addition of sulfur amino acids to the LPD (50 g protein daily), with amounts similar to that present in an HPD (150 g protein daily), caused an increase in urinary Ca of only 43% compared to HPD [124].

Calcium supplements are often available in the form of salts, with calcium carbonate and calcium citrate being the most popular. Other common forms of Ca include lactate and gluconate [125]. Calcium sources for these supplements include calcium carbonate ores, animal skeletons, seashells and crustaceans. However, natural calcium carbonate ores may contain harmful elements, such as heavy metals. Animal bones may carry a risk of prion transfer [126]. Therefore, other resources including egg shells or marine resources, have gained attention due to their high safety and biological activity in recent years. Świątkiewicz et al. [127] reported that Ca obtained from egg shells had a higher bioavailability compared to commercially available calcium carbonate. Similar results have been obtained in the study of Brennan et al. [128] using calcium-rich marine multimineral complex which significantly preserved trabecular bone microarchitecture and slowed the onset of bone loss in comparison with calcium carbonate. Omelka et al. [129] demonstrated the beneficial effects of co-administration of egg shell Ca with vitamins D3 and K2, as well as egg shell Ca with vitamin D3 only on the inhibition of bone loss in ovariectomized (OVX) rats. These two combinations significantly improved both biochemical and densitometric parameters consistent with osteoporosis.

#### 3.1.2. Phosphorus

Phosphorus is an essential micronutrient with various physiological roles. It is a component of nucleic acids, high-energy compounds (e.g., ATP, ADP, GTP, GDP), phospholipids and biological membranes, which plays an important role in energy metabolism, intracellular cell signaling and acid-base balance [130,131]. Phosphorus is the second basic component (after Ca) of bone tissue. The human body contains 550–770 g of P, of which almost 85% is stored in teeth and bones in the form of phosphoproteins and hydroxyapatite crystals [132]. Phosphorus deficiency causes sluggish growth and rickets in children and osteomalacia in adults. Lack of P in the diet is very rare in humans, due to its natural occurrence in large amounts of food and also the body’s high ability to absorb it. In healthy adults, the current RDA for P is 700 mg/day and 1250 mg/day during adolescent growth [130]. The impact of high P consumption on bone health is unequivocal. Some studies report that elevated P intake is detrimental to bone health in people whose dietary Ca:P ratio is extremely low [133,134]. On the contrary, there is a strong evidence that high P administration has no harmful effect on Ca balance in individuals with adequate Ca and P supplementation [135,136]. According to Lee and Cho [137], increased intake of P was associated with an improvement of 4.2% for BMC, 2.1% for BMD and reduced risk of osteoporosis by 45% in adult individuals whose Ca and P supplementation was within normal limits. Several animal studies have recorded that high dietary P levels, especially on a low Ca diet, decreased BMD through excessive parathyroid hormone (PTH) and osteopontin (OPN, bone matrix protein) excretion [131,138,139].

Phosphorus can be found in foods in naturally occurring forms, such as meat, dairy products and cereals, seeds, nuts, legumes, as well as in inorganic phosphate additives, which can be used for various purposes in food processing [130]. Some studies have shown that elevated P consumption from inorganic phosphate additives, such as cola, had detrimental effects on bone metabolism in adolescents and postmenopausal women [140,141]. On the contrary, Omelka et al. [142] did not observe any impact of long-term cola intake on the microstructure of cortical and trabecular bone tissues of adult mice using microcomputed tomography, probably due to a balanced diet and adequate physical activity. Cola as one of the most frequently consumed beverages today, supplies an amount of phosphoric acid that is easily absorbed. Moreover, cola can displace milk in the diet, so it can contribute to lower Ca and simultaneously higher inorganic P intake [143]. Based on current evidence, it would be desirable to limit the intake of phosphate additives.

#### 3.1.3. Magnesium

Magnesium is an essential micronutrient with a wide range of metabolic, regulatory and structural functions. It is the basis for ATP, regulates the activity of about 300 enzymes involved in the synthesis of proteins, carbohydrates and nucleic acids, and also maintains normal neuromuscular function [3,131]. Moreover, Mg stabilizes cell membranes, thereby reducing their permeability. Mg also antagonizes Ca and potassium (K) when it is absorbed in the small intestine, and chronically low levels of Ca and K may be related to the underlying Mg deficiency [144]. Bones store about 60% of total body Mg, approximately 30% is present in muscles, 9% in soft tissues, and 1% of Mg is found in extracellular fluids [145]. One third of skeletal Mg is located in the cortical bone, on the surface of hydroxyapatite crystals and in the areas around them [131]. Mg is important for bone development, as it stimulates bone formation and is also essential for bone mineralization [146].

Magnesium deficiency can have a detrimental effect on bone health directly (by enhancing osteoclast and reducing osteoblast activity, decreasing bone stiffness) and indirectly (by interfering with vitamin D and PTH, supporting inflammation and consequent bone loss) [3,116]. In animal studies, Mg deficiency was associated with easily broken bones, visible trabecular damages (microcracks), reduced cortical bone thickness and lower bone mechanical properties [147,148]. Orchard et al. [149] reported a decreased whole body and hip BMD in postmenopausal women with lower daily Mg intake. However, no relationship between low Mg supplementation and increased risk of fractures was determined in their study.

The effect of elevated Mg levels on bone quality remains controversial. According to Nieves [150], increased Mg intake was associated with more frequent wrist fractures in postmenopausal women, probably due to raising physical activity, as reported by the authors. Similarly, Orchard et al. [149] mention that the risk of wrist fractures increased with higher Mg administration. However, they also determined an elevated whole body and lumbar BMD (about 2% and 3%, respectively) in postmenopausal women who consumed >422.5 mg Mg/day compared to those consuming <206.5 mg Mg/day. The study by Houtkooper et al. [151] revealed a positive relationship between Mg intake and total BMD in premenopausal women simultaneously receiving Ca supplements. In postmenopausal women, an identical relationship between Mg administration and hip BMD (but not for BMD of radius) was noted [152]. In any case, further studies should be performed to clearly indicate that Mg supplementation may be beneficial in improving BMD.

The main sources of Mg are green vegetables (such as spinach), legumes, nuts, seeds, whole grains and almonds [22]. The current RDA of Mg is 320 mg/day for women and 420 mg/day for men [153]. Several factors, such as age, physical activity and smoking, can affect the concentration of Mg in the bones. In elderly people, serum Mg levels fall to 60–80% of those identified in children [154]. Insufficient physical activity was associated with decreased Mg levels in femoral neck samples [155]. Significantly reduced concentrations of Mg were found in the femoral head and trabecular bone of smokers in comparison with non-smokers [156,157]. In this sense, it is positive that seafood consumption can increase Mg level in the skeletal system [158]. Excessive intake of alcohol and coffee, inappropriate diet, stress and various diseases (e.g., diabetes, heart failure, hypertension, postmenopausal osteoporosis) can also adversely influence the content of Mg in the body.

### 3.2. Vitamins

#### 3.2.1. Vitamin D

Vitamin D (calciferol) is a fat-soluble vitamin that can be ingested as a food or as a dietary supplement (ergocalciferol—vitamin D2 or cholecalciferol—vitamin D3) or can be produced in the skin after sun exposure [144]. All aforementioned forms of vitamin D must then undergo two hydroxylation reactions (in the liver and kidneys) to being producing the biologically active form known as calcitriol—1,25 dihydroxyvitamin D (1,25(OH)2D) [159]. A total of 80–90% of vitamin D is reported to be achieved by dermal synthesis after sun exposure [160].

Vitamin D can directly and indirectly influence bone health [119]. It has the ability to regulate intestinal Ca absorption, bone and renal Ca resorption as well as PTH synthesis. Vitamin D also plays an important role in maintaining the optimal serum Ca and P levels and in skeletal mineralization [22,144]. In addition, vitamin D supplementation has a profound effect on bone and muscle strength [116], thereby reducing the risk of falls and subsequent fractures.

Foods that are high in vitamin D include egg yolk, fatty seafood, cod liver oil and breakfast cereals [21]. In accordance with the general recommendations, the optimal level of 1,25(OH)2D should be at least ≥20 ng/mL [161]. In individuals at higher risk of fractures, an elevated level of 1,25(OH)2D ≥30 ng/mL is recommended to maintain successful anti-osteoporotic treatment [162]. Although adequate dietary vitamin D intake is a key factor in preventing postmenopausal bone loss, it is difficult to obtain sufficient amounts only from one’s diet [118]. Due to a limited number of vitamin D-containing foods, its supplements are often needed to ensure appropriate intake [119].

Although many studies have reported a decrease in vertebral and non-vertebral fractures due to sufficient vitamin D supplementation in elderly patients [163,164,165], the meta-analyses by Boonen et al. [166], Chapuy et al. [167] and Bolland et al. [168] revealed that it did not alter the risk of osteoporosis. In the study of Burt et al. [169], high dose of vitamin D was consistent with reduced BMD in healthy adults without affecting bone strength. According to Alwan et al. [170], vitamin D was found to be positively related to trabecular bone score (TBS, an indicator of inner bone structural integrity) in healthy adults with adequate vitamin D intake (≥30 ng/mL, [170]). These findings explain data highlighting the benefits of vitamin D in preventing major vertebral and non-vertebral fractures in individuals with bone metabolic disorders such as osteoporosis or vitamin D deficiency [171]. Therefore, vitamin D supplementation, alone or in combination with Ca, appeared to be essential to enhance the positive effects of any specific therapy in such patients. In this context, simultaneous vitamin D and Ca intake in the treatment of osteoporosis is more effective than individual administrations [129,172,173].

Vitamin D deficiency can increase the risk of autoimmune diseases as well as non-skeletal chronic diseases and can also have a significant effect on the immune system, inflammation and muscle function [23]. In addition, low vitamin D levels are associated with an elevated risk of hip fractures in the elderly [119]. The study by LeBoff et al. [174] reported that 50% of postmenopausal women with a hip fracture had symptoms of vitamin D deficiency. Some anti-obesity drugs have been found to reduce the absorption of vitamin D [175]. High levels of vitamin A can also reduce the bioavailability of vitamin D by 30% [176].

Vitamin D deficiency is a well-known common feature in obese individuals [177], suggesting that adipose tissue may play a role in low serum vitamin D levels. It has been suggested that fat-soluble vitamin D could be sequestered in body fat stores, leading to its lower bioavailability in the obese state [178]. Therefore, high doses of vitamin D are needed to normalize serum vitamin D levels in obese individuals. According to Migliaccio et al. [179], vitamin D supplementation has some beneficial effects in the treatment of obesity and related comorbidities.

The safety of vitamin D therapy is mainly related to either dosage, type of supplementation, or in combination. Vitamin D3 boluses at doses >100,000 IU should not be used as they may cause an increased incidence of fractures and falls [180]. In any case, bodily self-regulation of vitamin D activation, effectively maintained using non-hydroxylated forms, minimizes the risk of toxicity [181]. Boluses and vitamin D hydroxy-analogs are not commonly recommended and should only be used in selected patients as both may cause hypercalcemia [162].

#### 3.2.2. Vitamin C

Vitamin C (ascorbic acid) is a water-soluble essential vitamin required for many physiological processes including biosynthesis of collagen, L-carnitine, hydroxyproline, hydroxylysine, several hormones (e.g., noradrenaline/adrenaline, peptide hormones), gene transcription, regulation of translation and elimination of tyrosine [112,182,183]. Vitamin C serves as an essential antioxidant, thus it can be used in the prevention of diseases associated with oxidative stress [184]. For this reason, it also plays an important role in immune responses [144]. Taking into account the bone, it is an essential cofactor not only for collagen production but also for osteoblast synthesis and differentiation and also has the ability to suppress osteoclast differentiation [185].

Vitamin C deficiency can lead to scurvy, which is manifested by osteolysis, osteonecrosis, decreased BMD, bone pain, impaired wound healing and pathological fractures [186,187]. Scurvy can eventually manifest as osteoporosis [188]. According to Doseděl et al. [183] and Fain [188], deficiency of vitamin C is associated with improper collagen synthesis, inappropriate osteoid formation and increased bone resorption. Several epidemiological studies have shown a negative relationship between vitamin C supplementation and fragility fracture risk (especially at the femoral neck and total hip) [144,186], demonstrating its therapeutic potential in the treatment of osteoporosis. Vitamin C intake is known to be consistent with increased gene expression for osteoblast differentiation, which stimulates osteoblast activity and accelerates bone formation [186]. In addition, ascorbic acid has a positive effect on alkaline phosphatase (ALP) activity. A strong correlation between vitamin C and BMD has also been established [79,112,189]. Significantly increased BMD of vertebrae and BMD of the spine and hip after vitamin C supplementation were determined in OVX rats and postmenopausal women, respectively [190,191]. This positive relationship was not confirmed in postmenopausal women in the Ahmadieh and Arabi study [192], suggesting the need for further research. Chuin et al. [193] revealed that antioxidant vitamins C and E may provide some protection against bone loss to the same extent as resistance exercise in elderly women.

The main sources of vitamin C are citrus fruits and juices, broccoli, tomato products, peppers, green leafy vegetables, potatoes, papaya, kiwi, strawberries and fortified breakfast cereals [112,182]. The recommended RDA for vitamin C is set at 75 mg/day for adult women and 90 mg/day for adult men [194]. However, the RDA of smokers increases by 35 mg/day due to elevated oxidative stress and metabolic turnover of vitamin C [195,196]. The harmful effect of ascorbic acid overdose on bone health remains unknown. Only mild symptoms such as osmotic diarrhea and related gastrointestinal disturbances are diagnosed because elevated vitamin C levels are usually excreted in the urine [197]. In this regard, there are concerns about possible urinary stone formation, since vitamin C increases oxalate levels in urine dose-dependently. Recent studies revealed that the risk of urinary stone formation seems to be very low after intake, even of high vitamin C doses [183]. In general, long-term raised urinary oxalate concentrations are required for stone formation, but elevated basal urinary oxalate levels can only be found in individuals at risk. According to Taylor et al. [198], oral intake of vitamin C in doses higher than 1 g increased the risk of stone formation by 41%. For this reason, the doses above 1 g should not be routinely recommended.

#### 3.2.3. Vitamin K

Vitamin K is a fat-soluble vitamin that occurs in two forms—as vitamin K1 (phylloquinone) and vitamin K2 (menaquinone). The first mentioned (K1) is the principal dietary form and can be achieved by consuming green vegetables, such as spinach, kale, broccoli, cauliflower, cabbage or supplements. The latter (K2) is produced by bacteria in the gut, but can also be found in fermented soy and dairy products (e.g., cheese), meat, fish, eggs, beef and pork liver. The current RDA is set at 90 µg/day for women and 120 µg/day for men [112,144]. Low vitamin K intake may be consistent with skeletal fragility. In the study by Feskanich et al. [199], such low administration increased the relative risk of hip fracture in both premenopausal and postmenopausal women.

Vitamin K regulates functions of osteocalcin, the most abundant non-collagenous protein within bone matrix, primarily produced by osteoblasts during their differentiation. After synthesis, osteocalcin undergoes carboxylation at glutamic acid residues by γ-glutamyl carboxylase and this process is facilitated by vitamin K. The carboxylated form of osteocalcin accumulates in bone matrix because of its strong affinity to hydroxyapatite. On the other hand, several studies revealed that uncarboxylated molecules of osteocalcin represent the bioactive form of this protein and they possess many metabolic functions including the regulation of glucose and energy metabolism [200,201]. Uncarboxylated or undercarboxylated osteocalcin promotes β-cell proliferation and insulin synthesis, increases the uptake of nutrients including glucose in skeletal muscle. There is also some evidence that osteocalcin affects muscle growth, fertility, brain development and cognition and anti-tumor immunity [202].

Vitamin K is also an important cofactor in enzymes involved in the synthesis of blood coagulation factors. According to Beulens et al. [203], higher vitamin K2 supplementation may be indirectly consistent with coronary artery calcification and subsequent cardiovascular disorder. On the contrary, increased dietary vitamin K1 intake does not have such an effect. Azuma and Inoue [204] reported that vitamin K status may also be related to muscle physical performance, and is not related to muscle strength.

Vitamin K2 is recognized as safe and effective in the treatment of age-related bone loss and osteoporosis [116]. It is involved in modulating the RANK/RANKL signaling pathway by inhibiting RANKL and reducing osteoclastogenesis [205]. In addition, vitamin K2 enhances osteoblastogenesis via the steroid and xenobiotic receptor, a nuclear receptor for osteoblasts, thereby promoting collagen accumulation [206].

The potential benefits of vitamin K2 supplementation for bone loss and BMD (mainly lumbar), especially in patients with osteoporosis, have been confirmed in several studies [207,208,209]. In non-osteoporotic individuals, no differences in BMD changes were reported [208]. Furthermore, its positive impact on fracture risk needs to be further demonstrated. According to Kanellakis et al. [210] and Omelka et al. [129], vitamin K2 in combination with Ca and vitamin D3 has the ability to improve BMD and reduce the risk of fractures in postmenopausal women and rats after ovariectomy, respectively, thus demonstrating its potential to enhance Ca and vitamin D3 treatment.

Several studies have shown interactions between vitamin K and postmenopausal therapies, which include vitamin E, Ca, estrogen and other hormones [22,79].

## 4. Flavonoid Polyphenols and Osteoporosis

Polyphenols (referred to as natural phytochemicals) are secondary metabolites of plants abundantly found in vegetables and fruits. They have a wide spectrum of biological activities, e.g., antioxidant, anti-inflammatory, anti-carcinogenic and antibacterial impacts [211]. Many studies have shown that a diet rich in polyphenols helps to delay the aging process and reduce the incidence of chronic diseases, such as cardiovascular disorders, arteriosclerosis, cancer, type 2 diabetes mellitus, cataracts, cognitive impairment, neurological diseases and even osteoporosis [19,107,212,213,214,215].

**Figure 2:**
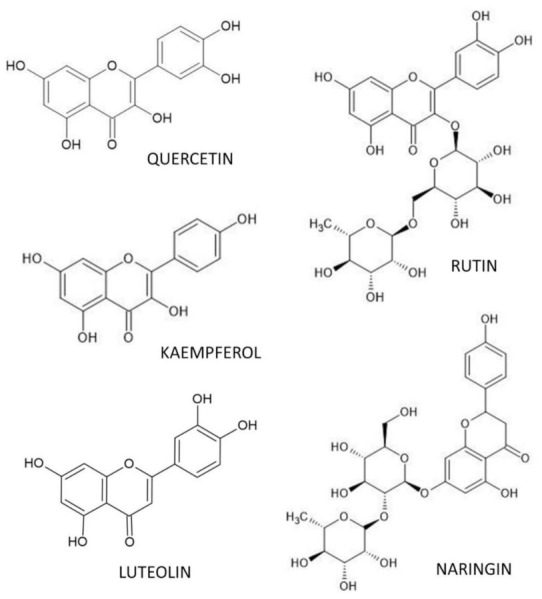
Chemical structures of described flavonoid polyphenols.

**Figure 3:**
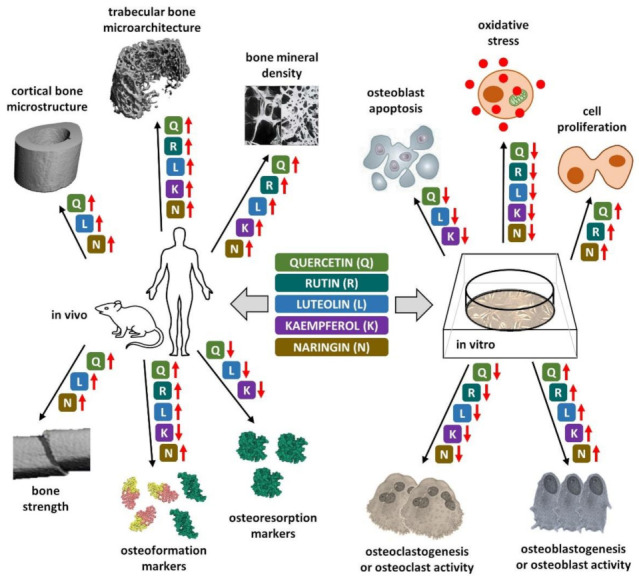
Effects of described flavonoid polyphenols on bone microstructural and biochemical parameters in vivo (left) and on bone cell parameters in vitro (right). The increase and decrease in a given parameter is indicated by red arrows pointing up and down, respectively. Q, quercetin; R, rutin; L, luteolin; K, kaempferol; N, naringin.

Flavonoids are a group of polyphenols widely distributed among vascular plants and are also omnipresent in the daily diet. Approximately 4000 natural products are known within the flavonoid family [216]. Many studies have reported that dietary flavonoid intake is closely related to reducing the risk of osteoporosis [217,218,219,220,221,222,223,224,225,226,227]. In our previous review [19], important non-flavonoid polyphenols (resveratrol, curcumin) as well as flavonoid polyphenols (genistein, daidzein, icariin, epigallocatechin gallate), which play a crucial role in skeletal health and prevention of osteoporosis were characterized. Other important flavonoids, such as quercetin, rutin, luteolin, kaempferol and naringin, with anti-osteoporotic impacts are described herein. Chemical structures of aforementioned flavonoid polyphenols are illustrated in Figure 2. Figure 3 shows their effects on bone-related parameters in vivo as well as on bone cell parameters in vitro.

### 4.1. Quercetin

Quercetin (3,5,7,3′,4′-pentahydroxyflavone) is a major dietary flavonoid in onion, red leaf lettuce, asparagus, green pepper, tomatoes and other vegetables, fruits and tea, as glycosides [227,228]. It has attracted great attention because of its anti-oxidative, anti-carcinogenic, anti-inflammatory, antibacterial, antiviral, anti-obesity, lipid-reducing and bone-conserving features [225,227]. Taking into account its protective effects against age-related bone loss, the in vivo study by Tsuji et al. [229] showed that dietary quercetin (2.5% for 4 weeks) can increase BMD and improve cortical and trabecular bone microstructure in OVX mice. Enhanced BMD, trabecular bone microarchitecture as well as improved bone strength have also been determined in OVX rats following quercetin administration (50 mg/kg/day for 8 weeks) [230]. In the study by Abd El-Fattah et al. [231], quercetin supplementation (50 mg/kg/day for 30 days) was associated with lower levels of ALP, acid phosphatase (ACP) and higher levels of serum Ca and P. Quercetin was also able to improve trabecular bone microarchitecture in disuse osteoporosis due to hind limb inactivity in mice. In addition, quercetin had a dual effect in promoting bone formation and inhibiting bone resorption, contributing to resistance to disuse-induced bone loss [227]. Numerous in vitro studies demonstrated an ability of quercetin to inhibit RANKL-induced osteoclastogenesis, osteoblast apoptosis and oxidative stress [227,232,233,234]. On the other hand, quercetin was able to elevate cell proliferation, bone sialoprotein (BSP), ALP activity, runt-related transcription factor 2 (Runx-2), Ca content when incubated with murine pre-osteoblastic MC3T3-E1 cells [235], rat osteoblast-like ROS 17/2.8 cells [236], osteoblasts derived from rat calvaria [237] and human osteoblast-like MG-63 cells [238]. In addition, quercetin is able to bind to estrogen receptors [239] and affects activity of both osteoblasts and osteoclasts as well as the expression and activity of various inflammatory cytokines involved in bone remodeling [240]. In the study by Wang et al. [241], quercetin enhanced osteogenic differentiation and antioxidant responses of bone mesenchymal stem cells by activating the AMPK/SIRT1 signaling pathway.

### 4.2. Rutin

Rutin (quercetin-3-rhamnosyl glucoside) is a flavonoid glycoside found in buckwheat, tobacco and rue and is also present in vegetables, fruits, tea, wine and herbs [242,243]. It is also known as vitamin P and possesses anti-oxidative, anti-viral, anti-inflammatory, anti-hypertensive, anti-carcinogenic and bone-protective properties [243,244]. Because rutin is a glycosylated form of quercetin, its clinical significance is limited by its low dissolution rate and oral bioavailability. Ovariectomized rats intragastrically administered with rutin (5 and 10 mg/kg for 3 months) had elevated femoral BMD, enhanced trabecular bone microarchitecture and lower levels of pro-inflammatory cytokines (e.g., IL-6, TNF-α and IFN-γ), pointing to a decreased osteoclast activity [221]. Rutin inhibited the expression of TNF-α and IL-6 also in the studies by Kyung et al. [245] and Middleton et al. [246]. Intraperitoneal administration of rutin (50 mg/kg/day for 4 weeks) significantly reduced osteoclast activity via inhibition of IL-1β, TNF-α and IL-6 in OVX mice as well [243]. Moreover, rutin significantly improved trabecular bone microarchitecture in the aforementioned study. Horcajada-Molteni et al. [247] found that rutin is able to inhibit OVX-induced osteopenia by increasing osteoblast activity, which is related to a higher level of osteocalcin in OVX rats. Gera et al. [222] used rutin nanosuspension (RUT-NS) as a potential therapy for osteoporosis. The authors determined elevated ALP activity and enhanced trabecular bone quality in OVX rats receiving RUT-NS (20 mg/kg for 2 months). Their findings from in vitro experiments with RUT-NS revealed increased cell proliferation, antioxidant activity and osteocalcin production in MG-63 osteoblast cells. In the study by Xiao et al. [248], administration of rutin (10 mg/kg/day for 10 weeks by gastric perfusion) partially reversed trabecular bone loss in rats after ovariectomy. In addition, rutin promoted bone marrow mesenchymal stem cell autophagy by inhibiting phosphorylated Akt in osteoporosis. Their results also suggest that rutin could regulate FNCD1 (encodes a fibronectin type III domain-containing protein) level and autophagy via the Akt/mTOR signaling pathway.

### 4.3. Luteolin

Luteolin (3,4,5,7-tetrahydroxyflavone) is a flavonoid present in many herbal extracts including celery, chamomile, green pepper, perilla leaf and seeds. It has a wide range of health benefits, including antioxidant, anti-inflammatory and anti-carcinogenic effects [249,250]. It effectively reduces the production of pro-inflammatory cytokines (e.g., TNF-α, IL-6) in an activated macrophage-like cell line [251]. An inhibited production of pro-inflammatory mediators by osteoblastic MC3T3-E1 cells has also been noted [252]. Luteolin supplementation (5 and 20 mg/kg/day for 30 days) significantly increased BMD, BMC, trabecular number in the femur of OVX mice and also prevented the decrease in bone strength [223]. Significantly elevated cortical BMD, cortical BMC, higher ALP levels, reduced osteocalcin and CTX levels were recorded in OVX mice as well [253]. The study by Jing et al. [224] showed that luteolin is able to alleviate glucocorticoid-induced osteoporosis by regulating the ERK/Lrp-5/GSK-3β signaling pathway in both in vivo and in vitro conditions. Luteolin treatment (25, 50 and 100 mg/kg/day for 2 months) was associated with higher trabecular number, improved trabecular bone microarchitecture, raised femoral BMD and BMC, promoted bone strength, reduced oxidative stress and increased osteoblastic differentiation in rats with glucocorticoid-induced osteoporosis. In dexamethasone (DXM)-treated MC3T3-E1 cells, luteolin administration (0.05, 0.1 and 0.2 µM for 48 h) elevated superoxide dismutase (SOD) activity and intracellular glutathione (GSH) level and decreased oxidative stress. Luteolin also promoted osteoblast differentiation via elevated expression levels of osteogenic markers as well as activation of the Wnt signaling pathway. In addition, increased ALP activity, promoted mineralization and reduced osteoclast activity via increasing OPG/RANKL ratio were demonstrated [224]. According to Kim et al. [223], luteolin inhibited osteoclastogenesis from bone marrow mononuclear cells and decreased resorption activity of mature osteoclasts via its inhibitory effects on RANKL-induced osteoclast formation [253].

### 4.4. Kaempferol

Kaempferol (3,5,7-trihydroxy-2-(4-hydroxyphenyl)-4H-1-benzopyran-4-one) is a flavonoid discovered in many vegetables, fruits and herbs, including spinach, kale, broccoli, tomatoes, grapes, tea and *Ginkgo biloba* leaves [254]. Kaempferol possesses various health benefits, which include cardioprotective, neuroprotective, anti-allergic, anti-carcinogenic, anti-microbial, anti-obesity, anti-oxidative, anti-inflammatory and anti-osteoporotic impacts [217,255]. Various studies have shown that kaempferol promotes bone formation and induces bone cell differentiation to alleviate osteoporosis [217,218]. In the study by Trivedi et al. [256], kaempferol supplementation (5 mg/kg/bw for 10 weeks) was associated with raised femoral BMD, compressive energy in vertebrae and bone turnover inhibition accompanied by decreased serum ALP levels in OVX rats. Moreover, kaempferol increased mineralized nodules in rat primary osteoblasts and inhibited bone marrow adipogenesis. Increased femoral BMD has also been observed in OVX rats after kaempferol treatment (5 mg/kg/bw for 8 weeks) in the study by Nowak et al. [257]. These authors determined improved trabecular bone microarchitecture, decreased levels of bone turnover markers (osteocalcin, CTX) as well as reduced RANKL level. The results of Liu et al. [258] correspond to the aforementioned studies, as they again point to an elevated BMD and relative bone volume in OVX rats following kaempferol administration (5 mg/kg/day for 12 weeks). In addition, kaempferol promoted the differentiation of bone marrow mesenchymal stem cells by raising the expression of CTCL12, indicating its ability to alleviate osteoporosis. Several in vitro studies have shown anti-osteoclastogenic impacts of kaempferol that may be related to downregulation of osteoclastogenic factors including RANKL, Fos proto-oncogene (c-Fos), nuclear factor of activated T-cells cytoplasmic 1 (NFATc1) and tumor necrosis factor receptor-associated factor 6 (TRAF6) in kaempferol-treated cells [219,259]. Moreover, kaempferol can also modulate bone metabolism through estrogen receptor (ER) [260]. Studies by Tang et al. [261] and Yang et al. [262] confirmed that kaempferol activated ERβ-mediated ERE-reporter transcription in MG-63 osteoblasts and osteoblast-like UMR cells, respectively. Stimulation of estrogen signaling is associated with activation of the Wnt signaling pathway, thereby achieving the potential for bone-protective effects [218].

### 4.5. Naringin

Naringin (naringenin 7-O-neohesperidose) is a flavanone glycoside commonly found in citrus fruits, grapes, cherries, tomatoes and oregano. It has a strong hot taste of grapefruit juice [263,264]. Naringin possesses a number of biological and pharmacological characteristics, such as antioxidant, anti-carcinogenic, anti-inflammatory, anti-ulcer, anti-apoptic and anti-osteoporotic effects [265]. Naringin supplementation elevated femoral bone mass by increasing both trabecular and cortical bone quality in healthy mice [220]. In the study by Pang et al. [266], enhanced femoral, tibial and lumbal bone quality as well as reduced urinary Ca excretion and higher bone strength were determined in OVX mice after naringin treatment (200 and 400 mg/kg/day for 6 weeks). Wang et al. [267] reported raised bone strength in OVX mice even at a lower dose of naringin (5 mg/kg for 6 weeks), while the authors noted improved ALP, RUNX2 and collagen I expression in vivo as well. Significantly increased BMD and enhanced trabecular bone microarchitecture were observed in OVX mice following naringin administration (300 mg/kg for 2 months) [268]. Naringin intake was consistent with identical findings also in OVX rats [269]. Various in vitro studies indicate that naringin significantly elevated proliferation of osteoprogenitor cells, including murine pre-osteoblasts (MC3T3-E1) as well as cells with an osteoblastic phenotype, such as human and murine primary fetal osteoblasts [226,270]. Naringin administration raised in vitro expression of bone morphogenetic proteins (BMPs) and activation of Wnt/β-catenin pathway [264]. Moreover, naringin exerts estrogen-like effect and significantly elevates ALP activity in rat UMR-106 cells [266,271]. Naringin was also able to inhibit osteoclastogenesis by modifying RANK/RANKL interactions and inducing apoptosis in osteoclasts [264].

## 5. Conclusions

Adequate nutritional status is crucial for skeletal health. A balanced diet that meets daily caloric needs and contains the required daily intake of Ca and vitamin D is a key factor in achieving maximum peak bone mass as well as reducing the rate of bone loss in the elderly. The amount of other nutrients in food (including both macro- and micronutrients) can also affect bone health. It should be noted that there is no accidental relationship between different nutrients and osteoporosis prevention and treatment with the exception of Ca and vitamin D. The lack of these two nutrients causes a higher risk of fragility fractures. The impacts of Ca and vitamin D on osteoporotic bone quality cannot be assessed separately from other components of the diet such as P, Mg and vitamins C and K, which are involved in bone metabolism. Therefore, these nutrients appear to be promising for the prevention and treatment of osteoporosis as well; however, further experiments and data are required. There are also a large number of flavonoid polyphenols, which are considered vital elements in reducing the risk of osteoporosis. Diets mainly rich in fruits, vegetables, seafood and fish oil supplements and specific dairy products contain all these nutritional substances and are considered healthy for bones. Thus, in addition to pharmacotherapy, this healthy diet can have an anti-osteoporotic impact (it may slow the degeneration of bone and muscle tissue and thereby reduce the risk of falls and fractures) together with regular physical activity. However, the effect of diet becomes more important with increasing age and, in addition, may depend on an individual’s genotype, gene-diet interactions or the composition and function of the gut microbiota.

Based on the information provided above, the following dietary recommendations can be made to help prevent osteoporosis:

- Foods with a high energy density, such as foods rich in PUFAs, fruits and vegetables, high in fibers and high-quality animal or plant-based proteins, should be selected as a matter of priority to ensure sufficient vitamins and minerals.
- Supplements, such as calcium carbonate or calcium citrate, may be used to improve skeletal health if there are dietary deficiencies.
- Vitamin D deficiency can be corrected by either extending time spent outdoors, taking supplements, or in combination.
- Foods and beverages with a poor nutrient density, such as foods made from simple carbohydrates, carbonated and sugar-sweetened beverages or products high in Na or SFAs should be either reduced or excluded.

## Figures and Tables

**Table 1:** The amount of described macronutrients and micronutrients in different types of foods.

Type of Food	Amount of Nutrients per 100 g
Proteins (g) [24]	
meat (beef, pork, chicken)	15.43–20.04
sea fish	18.88
pike, trout	11.00
oatmeal	13.10
eggs	12.40
dairy products (yogurt, cheese, cream cheese)	4.40–26.00
legumes (chickpeas, beans, peas, lentils)	20–24.20
tofu	7.80
soy	35.40
cocoa powder	22.70
nuts (almonds, peanuts, pistachios)	19.70–25.80
seeds (poppy, sesame, pumpkins)	20.40–24.50
Saturated fatty acids (g) [25,26]	
milk fat	63.40
coconut oil	86.50
palm kernel oil	81.50
palm oil	49.30
cocoa butter	59.70
Monounsaturated fatty acids (g) [25,26]	
milk fat	25.90
palm oil	37.00
cocoa butter	32.90
olive oil	73.00
soybean oil	22.70
high linoleic acid sunflower oil	19.50
Polyunsaturated fatty acids (g) [25,26]	
palm oil	9.30
olive oil	10.50
soybean oil	57.30
high linoleic acid sunflower oil	65.70
Carbohydrates (g) [24]	
oatmeal	68.10
wheat flour	73.10
wheat white bread	50.80
legumes (chickpeas, beans, peas, lentils)	58.00–60.50
fruits (blackcurrant, grapes, bananas)	17.20–21.80
vegetables (potatoes, sweet corn, garlic)	18.80–25.00
nuts (peanuts, pistachios, chestnuts)	18.20–53.00
curry spice	61.80
black tea	55.70
bitter chocolate	117.12
Calcium (mg) [25,26]	
cow milk (natural)	119.14
hard cheese	981.71–1218.00
soft cheese	732.86
eggs	57.06
marjoram	1388.00
poppy seeds (natural)	1513.71
salmon (Atlantic)	20.00
almonds	229.71
green-leaf vegetables (head cabbage, curly kale)	47.50–163.57
legume-based dishes	30.91
tofu	162.43
seafood-based dishes	38.05
mineral water rich in calcium (250 mL)	100.00
Phosphorus (mg) [25,26]	
meat (chicken, duck, turkey, goat)	188.57–234.86
cow milk	92.86
hard cheese	786.86
soft cheese	345.57
cereals and cereal-like grains	271.00
seeds (linseed, pumpkin, sesame, sunflower, poppy)	603.00–861.71
nuts (cashew, peanuts, walnuts)	519.57–369.71
legume-based dishes	132.37
Magnesium (mg) [25,26]	
spinach	61.99
legume-based dishes	41.20
nuts (walnuts, hazelnuts, peanuts, almonds, cashew)	150.71–262.14
seeds (linseed, pumpkin, sesame, sunflower)	272.00–358.83
grain-based dishes	100.00
bitter chocolate	164.29
Vitamin D (μg) [27,28]	
D2/D3 in soybean oil	700.00
D2/D3 in sunflower oil	11.20–14.50
D2 in dry mushroom powder	4420.00
eggs	3.20
fishes (mackerel, salmon, sardines, tuna)	3.20–8.00
cod	trace
Vitamin C (mg) [29,30,31]	
citrus fruits (lemon, orange, grapefruit) products	30.00–53.00
broccoli	34.80–93.10
tomato products	12.00
peppers (red peppers, chili peppers)	190.00–245.00
green leafy vegetables (spinach, cabbage, kale, cauliflower)	30.00–48.00
potatoes	25.00
papaya	61.00
kiwifruit	93.00
red current	80.00
strawberry and its products	54.00–60.00
Vitamin K (μg) [25,26]	
dark green leafy vegetables (spinach, curly kale)	362.50–817.00
fruits (kiwifruit, blackcurrants, prunes, rose hip)	25.00–92.00
chickpea	264.00
liver (beef, chicken, veal)	75.00–89.00
parsley	488.75

## Data Availability

Data sharing not applicable.

## References

[1] Kanis J.A., Cooper C., Rizzoli R., Reginster J.-Y. (2019). Scientific Advisory Board of the European Society for Clinical and Economic Aspects of Osteoporosis (ESCEO) and the Committees of Scientific Advisors and National Societies of the International Osteoporosis Foundation (IOF) European Guidance for the Diagnosis and Management of Osteoporosis in Postmenopausal Women. Osteoporos. Int..

[2] Rachner T.D., Khosla S., Hofbauer L.C. (2011). New Horizons in Osteoporosis. Lancet.

[3] Castiglioni S., Cazzaniga A., Albisetti W., Maier J.A.M. (2013). Magnesium and Osteoporosis: Current State of Knowledge and Future Research Directions. Nutrients.

[4] Ström O., Borgström F., Kanis J.A., Compston J., Cooper C., McCloskey E.V., Jönsson B. (2011). Osteoporosis: Burden, Health Care Provision and Opportunities in the EU: A Report Prepared in Collaboration with the International Osteoporosis Foundation (IOF) and the European Federation of Pharmaceutical Industry Associations (EFPIA). Arch. Osteoporos..

[5] Bonjour J.-P., Guéguen L., Palacios C., Shearer M.J., Weaver C.M. (2009). Minerals and Vitamins in Bone Health: The Potential Value of Dietary Enhancement. Br. J. Nutr..

[6] Warensjö Lemming E., Byberg L. (2020). Is a Healthy Diet Also Suitable for the Prevention of Fragility Fractures?. Nutrients.

[7] Prentice A. (2001). The Relative Contribution of Diet and Genotype to Bone Development. Proc. Nutr. Soc..

[8] Ilesanmi-Oyelere B.L., Kruger M.C. (2020). Nutrient and Dietary Patterns in Relation to the Pathogenesis of Postmenopausal Osteoporosis-A Literature Review. Life.

[9] Compston J.E., McClung M.R., Leslie W.D. (2019). Osteoporosis. Lancet.

[10] Das U.N. (2013). Catechins and Osteoporosis. Nutrition.

[11] Goltzman D., Rhim J.S., Dritschilo A., Kremer R. (2019). The Aging Skeleton. Human Cell Transformation: Advances in Cell Models for the Study of Cancer and Aging.

[12] Rosen C.J., Bouxsein M.L. (2006). Mechanisms of Disease: Is Osteoporosis the Obesity of Bone?. Nat. Clin. Pract. Rheumatol..

[13] Corrado A., Cici D., Rotondo C., Maruotti N., Cantatore F.P. (2020). Molecular Basis of Bone Aging. Int. J. Mol. Sci..

[14] Chandra A., Rajawat J. (2021). Skeletal Aging and Osteoporosis: Mechanisms and Therapeutics. Int. J. Mol. Sci..

[15] Cannata-Andía J.B., Carrillo-López N., Messina O.D., Hamdy N.A.T., Panizo S., Ferrari S.L., On behalf of the International Osteoporosis Foundation (IOF) (2021). Working Group on Bone and Cardiovascular Diseases Pathophysiology of Vascular Calcification and Bone Loss: Linked Disorders of Ageing?. Nutrients.

[16] Kearns A.E., Khosla S., Kostenuik P.J. (2008). Receptor Activator of Nuclear Factor KappaB Ligand and Osteoprotegerin Regulation of Bone Remodeling in Health and Disease. Endocr. Rev..

[17] Eghbali-Fatourechi G., Khosla S., Sanyal A., Boyle W.J., Lacey D.L., Riggs B.L. (2003). Role of RANK Ligand in Mediating Increased Bone Resorption in Early Postmenopausal Women. J. Clin. Investig..

[18] Hejazi J., Davoodi A., Khosravi M., Sedaghat M., Abedi V., Hosseinverdi S., Ehrampoush E., Homayounfar R., Shojaie L. (2020). Nutrition and Osteoporosis Prevention and Treatment. Biomed. Res. Ther..

[19] Martiniakova M., Babikova M., Omelka R. (2020). Pharmacological Agents and Natural Compounds: Available Treatments for Osteoporosis. J. Physiol. Pharmacol..

[20] Kim B., Cho Y.J., Lim W. (2021). Osteoporosis Therapies and Their Mechanisms of Action (Review). Exp. Ther. Med..

[21] Karpouzos A., Diamantis E., Farmaki P., Savvanis S., Troupis T. (2017). Nutritional Aspects of Bone Health and Fracture Healing. J. Osteoporos..

[22] Ratajczak A.E., Rychter A.M., Zawada A., Dobrowolska A., Krela-Kaźmierczak I. (2020). Nutrients in the Prevention of Osteoporosis in Patients with Inflammatory Bowel Diseases. Nutrients.

[23] Lorincz C., Manske S.L., Zernicke R. (2009). Bone Health: Part 1, Nutrition. Sports Health.

[24] EuroFIR—European Food Information Resource. https://www.eurofir.org/.

[25] Food Composition Data|EFSA. https://www.efsa.europa.eu/en/microstrategy/food-composition-data.

[26] EFSA Panel on Dietetic Products, Nutrition, and Allergies (NDA) (2010). Scientific Opinion on Dietary Reference Values for Fats, Including Saturated Fatty Acids, Polyunsaturated Fatty Acids, Monounsaturated Fatty Acids, Trans Fatty Acids, and Cholesterol. EFSA J..

[27] Lavelli V., D’Incecco P., Pellegrino L. (2021). Vitamin D Incorporation in Foods: Formulation Strategies, Stability, and Bioaccessibility as Affected by the Food Matrix. Foods.

[28] Spiro A., Buttriss J.L. (2014). Vitamin D: An Overview of Vitamin D Status and Intake in Europe. Nutr. Bull..

[29] Favell D.J. (1998). A Comparison of the Vitamin C Content of Fresh and Frozen Vegetables. Food Chem..

[30] Szeto Y.T., Tomlinson B., Benzie I.F.F. (2002). Total Antioxidant and Ascorbic Acid Content of Fresh Fruits and Vegetables: Implications for Dietary Planning and Food Preservation. Br. J. Nutr..

[31] Giannakourou M.C., Taoukis P.S. (2021). Effect of Alternative Preservation Steps and Storage on Vitamin C Stability in Fruit and Vegetable Products: Critical Review and Kinetic Modelling Approaches. Foods.

[32] Heaney R.P., Layman D.K. (2008). Amount and Type of Protein Influences Bone Health. Am. J. Clin. Nutr..

[33] Heaney R.P. (2002). Protein and Calcium: Antagonists or Synergists?. Am. J. Clin. Nutr..

[34] Medicine I. (2005). Dietary Reference Intakes for Energy, Carbohydrate, Fiber, Fat, Fatty Acids, Cholesterol, Protein, and Amino Acids.

[35] Richter M., Baerlocher K., Bauer J.M., Elmadfa I., Heseker H., Leschik-Bonnet E., Stangl G., Volkert D., Stehle P. (2019). Revised Reference Values for the Intake of Protein. Ann. Nutr. Metab..

[36] Wallace T.C., Frankenfeld C.L. (2017). Dietary Protein Intake above the Current RDA and Bone Health: A Systematic Review and Meta-Analysis. J. Am. Coll. Nutr..

[37] Promislow J.H.E., Goodman-Gruen D., Slymen D.J., Barrett-Connor E. (2002). Protein Consumption and Bone Mineral Density in the Elderly: The Rancho Bernardo Study. Am. J. Epidemiol..

[38] Coin A., Perissinotto E., Enzi G., Zamboni M., Inelmen E.M., Frigo A.C., Manzato E., Busetto L., Buja A., Sergi G. (2008). Predictors of Low Bone Mineral Density in the Elderly: The Role of Dietary Intake, Nutritional Status and Sarcopenia. Eur. J. Clin. Nutr..

[39] Kerstetter J.E., Looker A.C., Insogna K.L. (2000). Low Dietary Protein and Low Bone Density. Calcif. Tissue Int..

[40] Bonjour J.-P. (2016). The Dietary Protein, IGF-I, Skeletal Health Axis. Horm. Mol. Biol. Clin. Investig..

[41] Darling A.L., Millward D.J., Lanham-New S.A. (2021). Dietary Protein and Bone Health: Towards a Synthesised View. Proc. Nutr. Soc..

[42] Deane C.S., Bass J.J., Crossland H., Phillips B.E., Atherton P.J. (2020). Animal, Plant, Collagen and Blended Dietary Proteins: Effects on Musculoskeletal Outcomes. Nutrients.

[43] Conigrave A.D., Brown E.M., Rizzoli R. (2008). Dietary Protein and Bone Health: Roles of Amino Acid-Sensing Receptors in the Control of Calcium Metabolism and Bone Homeostasis. Annu. Rev. Nutr..

[44] Itkonen S.T., Päivärinta E., Pellinen T., Viitakangas H., Risteli J., Erkkola M., Lamberg-Allardt C., Pajari A.-M. (2021). Partial Replacement of Animal Proteins with Plant Proteins for 12 Weeks Accelerates Bone Turnover Among Healthy Adults: A Randomized Clinical Trial. J. Nutr..

[45] Liu Z., Jeppesen P.B., Gregersen S., Chen X., Hermansen K. (2008). Dose- and Glucose-Dependent Effects of Amino Acids on Insulin Secretion from Isolated Mouse Islets and Clonal INS-1E Beta-Cells. Rev. Diabet. Stud..

[46] Yang J., Zhang X., Wang W., Liu J. (2010). Insulin Stimulates Osteoblast Proliferation and Differentiation through ERK and PI3K in MG-63 Cells. Cell Biochem. Funct..

[47] Jennings A., MacGregor A., Spector T., Cassidy A. (2016). Amino Acid Intakes Are Associated With Bone Mineral Density and Prevalence of Low Bone Mass in Women: Evidence From Discordant Monozygotic Twins. J. Bone Miner. Res..

[48] Fini M., Torricelli P., Giavaresi G., Carpi A., Nicolini A., Giardino R. (2001). Effect of L-Lysine and L-Arginine on Primary Osteoblast Cultures from Normal and Osteopenic Rats. Biomed. Pharmacother..

[49] Iguacel I., Miguel-Berges M.L., Gómez-Bruton A., Moreno L.A., Julián C. (2019). Veganism, Vegetarianism, Bone Mineral Density, and Fracture Risk: A Systematic Review and Meta-Analysis. Nutr. Rev..

[50] Shams-White M.M., Chung M., Fu Z., Insogna K.L., Karlsen M.C., LeBoff M.S., Shapses S.A., Sackey J., Shi J., Wallace T.C. (2018). Animal versus Plant Protein and Adult Bone Health: A Systematic Review and Meta-Analysis from the National Osteoporosis Foundation. PLoS ONE.

[51] Sahni S., Cupples L.A., Mclean R.R., Tucker K.L., Broe K.E., Kiel D.P., Hannan M.T. (2010). Protective Effect of High Protein and Calcium Intake on the Risk of Hip Fracture in the Framingham Offspring Cohort. J. Bone Miner. Res..

[52] Castaneda C. (2002). Muscle Wasting and Protein Metabolism1. J. Anim. Sci..

[53] Bettis T., Kim B.-J., Hamrick M.W. (2018). Impact of Muscle Atrophy on Bone Metabolism and Bone Strength: Implications for Muscle-Bone Crosstalk with Aging and Disuse. Osteoporos. Int..

[54] Verschueren S., Gielen E., O’Neill T.W., Pye S.R., Adams J.E., Ward K.A., Wu F.C., Szulc P., Laurent M., Claessens F. (2013). Sarcopenia and Its Relationship with Bone Mineral Density in Middle-Aged and Elderly European Men. Osteoporos. Int..

[55] Kim S., Won C.W., Kim B.S., Choi H.R., Moon M.Y. (2014). The Association between the Low Muscle Mass and Osteoporosis in Elderly Korean People. J. Korean Med. Sci..

[56] Chen Y., Chen J., Chen J., Yu H., Zheng Y., Zhao J., Zhu J. (2020). Recent Advances in Seafood Bioactive Peptides and Their Potential for Managing Osteoporosis. Crit. Rev. Food Sci. Nutr..

[57] Bhandari D., Rafiq S., Gat Y., Gat P., Waghmare R., Kumar V. (2020). A Review on Bioactive Peptides: Physiological Functions, Bioavailability and Safety. Int. J. Pept. Res. Ther..

[58] Harnedy P.A., FitzGerald R.J. (2012). Bioactive Peptides from Marine Processing Waste and Shellfish: A Review. J. Funct. Foods.

[59] Kim S.-K., Mendis E. (2006). Bioactive Compounds from Marine Processing Byproducts—A Review. Food Res. Int..

[60] Zdzieblik D., Oesser S., König D. (2021). Specific Bioactive Collagen Peptides in Osteopenia and Osteoporosis: Long-Term Observation in Postmenopausal Women. J. Bone Metab..

[61] Reddi S., Mada S., Kumar N., Kumar R., Ahmad N., Karvande A., Kapila S., Kapila R., Trivedi R. (2019). Antiosteopenic Effect of Buffalo Milk Casein-Derived Peptide (NAVPITPTL) in Ovariectomized Rats. Int. J. Pept. Res. Ther..

[62] Cicero A.F.G., Fogacci F., Colletti A. (2017). Potential Role of Bioactive Peptides in Prevention and Treatment of Chronic Diseases: A Narrative Review. Br. J. Pharmacol..

[63] Arab L. (2003). Biomarkers of Fat and Fatty Acid Intake. J. Nutr..

[64] Bajželj B., Laguzzi F., Röös E. (2021). The Role of Fats in the Transition to Sustainable Diets. Lancet Planet. Health.

[65] Loef M., Schoones J.W., Kloppenburg M., Ioan-Facsinay A. (2019). Fatty Acids and Osteoarthritis: Different Types, Different Effects. Jt. Bone Spine.

[66] Grundy S.M., Caballero B. (2013). Cholesterol: Factors Determining Blood Levels. Encyclopedia of Human Nutrition.

[67] Degirolamo C., Rudel L.L. (2010). Dietary Monounsaturated Fatty Acids Appear Not to Provide Cardioprotection. Curr. Atheroscler. Rep..

[68] Kuna A., Achinna P. (2013). Mono Unsaturated Fatty Acids for CVD and Diabetes: A Healthy Choice. Int. J. Nutr. Pharmacol. Neurol. Dis..

[69] Abdullah M.M.H., Jew S., Jones P.J.H. (2017). Health Benefits and Evaluation of Healthcare Cost Savings If Oils Rich in Monounsaturated Fatty Acids Were Substituted for Conventional Dietary Oils in the United States. Nutr. Rev..

[70] Schwingshackl L., Hoffmann G. (2014). Monounsaturated Fatty Acids, Olive Oil and Health Status: A Systematic Review and Meta-Analysis of Cohort Studies. Lipids Health Dis..

[71] Del Gobbo L.C., Imamura F., Aslibekyan S., Marklund M., Virtanen J.K., Wennberg M., Yakoob M.Y., Chiuve S.E., Dela Cruz L., Frazier-Wood A.C. (2016). ω-3 Polyunsaturated Fatty Acid Biomarkers and Coronary Heart Disease: Pooling Project of 19 Cohort Studies. JAMA Intern. Med..

[72] Kato I., Toniolo P., Zeleniuch-Jacquotte A., Shore R.E., Koenig K.L., Akhmedkhanov A., Riboli E. (2000). Diet, Smoking and Anthropometric Indices and Postmenopausal Bone Fractures: A Prospective Study. Int. J. Epidemiol..

[73] Romero Márquez J.M., Varela López A., Navarro Hortal M.D., Badillo Carrasco A., Quiles Morales J.L. (2021). Molecular Interactions between Dietary Lipids and Bone Tissue during Aging. Int. J. Mol. Sci..

[74] Corwin R.L., Hartman T.J., Maczuga S.A., Graubard B.I. (2006). Dietary Saturated Fat Intake Is Inversely Associated with Bone Density in Humans: Analysis of NHANES III. J. Nutr..

[75] Orchard T.S., Cauley J.A., Frank G.C., Neuhouser M.L., Robinson J.G., Snetselaar L., Tylavsky F., Wactawski-Wende J., Young A.M., Lu B. (2010). Fatty Acid Consumption and Risk of Fracture in the Women’s Health Initiative1234. Am. J. Clin. Nutr..

[76] García-Martínez O., Rivas A., Ramos-Torrecillas J., De Luna-Bertos E., Ruiz C. (2014). The Effect of Olive Oil on Osteoporosis Prevention. Int. J. Food Sci. Nutr..

[77] Roncero-Martín R., Aliaga Vera I., Moreno-Corral L.J., Moran J.M., Lavado-Garcia J.M., Pedrera-Zamorano J.D., Pedrera-Canal M. (2018). Olive Oil Consumption and Bone Microarchitecture in Spanish Women. Nutrients.

[78] Farina E.K., Kiel D.P., Roubenoff R., Schaefer E.J., Cupples L.A., Tucker K.L. (2011). Protective Effects of Fish Intake and Interactive Effects of Long-Chain Polyunsaturated Fatty Acid Intakes on Hip Bone Mineral Density in Older Adults: The Framingham Osteoporosis Study. Am. J. Clin. Nutr..

[79] Sahni S., Mangano K.M., McLean R.R., Hannan M.T., Kiel D.P. (2015). Dietary Approaches for Bone Health: Lessons from the Framingham Osteoporosis Study. Curr. Osteoporos. Rep..

[80] Tartibian B., Hajizadeh Maleki B., Kanaley J., Sadeghi K. (2011). Long-Term Aerobic Exercise and Omega-3 Supplementation Modulate Osteoporosis through Inflammatory Mechanisms in Post-Menopausal Women: A Randomized, Repeated Measures Study. Nutr. Metab..

[81] Farina E.K., Kiel D.P., Roubenoff R., Schaefer E.J., Cupples L.A., Tucker K.L. (2011). Dietary Intakes of Arachidonic Acid and α-Linolenic Acid Are Associated with Reduced Risk of Hip Fracture in Older Adults. J. Nutr..

[82] Longo A.B., Ward W.E. (2016). PUFAs, Bone Mineral Density, and Fragility Fracture: Findings from Human Studies. Adv. Nutr..

[83] Abou-Saleh H., Ouhtit A., Halade G.V., Rahman M.M. (2019). Bone Benefits of Fish Oil Supplementation Depend on Its EPA and DHA Content. Nutrients.

[84] Kim S., Henneicke H., Caavanagh L.L., Macfarlane E., Thai L.J., Foong D., Gasparini S.J., Fong-Yee C., Swarbrick M.M., Seibel M.J. (2021). Osteoblastic Glucocorticoid Signaling Exacerbates High-Fat-Diet- Induced Bone Loss and Obesity. Bone Res..

[85] Macdonald H.M., New S.A., Golden M.H., Grubb D.A., Reid D.M. (2001). Food Groups Affecting Perimenopausal and Early Postmenopausal Bone Loss in Scottish Women. Nutr. Asp. Osteoporos..

[86] Turcotte A.-F., O’Connor S., Morin S.N., Gibbs J.C., Willie B.M., Jean S., Gagnon C. (2021). Association between Obesity and Risk of Fracture, Bone Mineral Density and Bone Quality in Adults: A Systematic Review and Meta-Analysis. PLoS ONE.

[87] Walsh J.S., Vilaca T. (2017). Obesity, Type 2 Diabetes and Bone in Adults. Calcif. Tissue Int..

[88] Cao J.J. (2011). Effects of Obesity on Bone Metabolism. J. Orthop. Surg. Res..

[89] Rinonapoli G., Pace V., Ruggiero C., Ceccarini P., Bisaccia M., Meccariello L., Caraffa A. (2021). Obesity and Bone: A Complex Relationship. Int. J. Mol. Sci..

[90] Ben-Porat T., Elazary R., Sherf-Dagan S., Goldenshluger A., Brodie R., Mintz Y., Weiss R. (2018). Bone Health Following Bariatric Surgery: Implications for Management Strategies to Attenuate Bone Loss. Adv. Nutr..

[91] Shapses S.A., Sukumar D. (2012). Bone Metabolism in Obesity and Weight Loss. Annu. Rev. Nutr..

[92] Kiely L.J., Hickey R.M. (2022). Characterization and Analysis of Food-Sourced Carbohydrates. Methods Mol. Biol..

[93] Soliman G.A. (2019). Dietary Fiber, Atherosclerosis, and Cardiovascular Disease. Nutrients.

[94] Cohen T.R., Hazell T.J., Vanstone C.A., Plourde H., Rodd C.J., Weiler H.A. (2013). A Family-Centered Lifestyle Intervention to Improve Body Composition and Bone Mass in Overweight and Obese Children 6 through 8 Years: A Randomized Controlled Trial Study Protocol. BMC Public Health.

[95] Tjäderhane L., Larmas M. (1998). A High Sucrose Diet Decreases the Mechanical Strength of Bones in Growing Rats. J. Nutr..

[96] Terada M., Inaba M., Yano Y., Hasuma T., Nishizawa Y., Morii H., Otani S. (1998). Growth-Inhibitory Effect of a High Glucose Concentration on Osteoblast-like Cells. Bone.

[97] Ericsson Y., Angmar-Månsson B., Flores M. (1990). Urinary Mineral Ion Loss after Sugar Ingestion. Bone Miner..

[98] Douard V., Sabbagh Y., Lee J., Patel C., Kemp F.W., Bogden J.D., Lin S., Ferraris R.P. (2013). Excessive Fructose Intake Causes 1,25-(OH)(2)D(3)-Dependent Inhibition of Intestinal and Renal Calcium Transport in Growing Rats. Am. J. Physiol. Endocrinol. Metab..

[99] Tsanzi E., Light H.R., Tou J.C. (2008). The Effect of Feeding Different Sugar-Sweetened Beverages to Growing Female Sprague-Dawley Rats on Bone Mass and Strength. Bone.

[100] Ogur R., Uysal B., Ogur T., Yaman H., Oztas E., Ozdemir A., Hasde M. (2007). Evaluation of the Effect of Cola Drinks on Bone Mineral Density and Associated Factors. Basic Clin. Pharmacol. Toxicol..

[101] Vartanian L.R., Schwartz M.B., Brownell K.D. (2007). Effects of Soft Drink Consumption on Nutrition and Health: A Systematic Review and Meta-Analysis. Am. J. Public Health.

[102] Jakeman S.A., Henry C.N., Martin B.R., McCabe G.P., McCabe L.D., Jackson G.S., Peacock M., Weaver C.M. (2016). Soluble Corn Fiber Increases Bone Calcium Retention in Postmenopausal Women in a Dose-Dependent Manner: A Randomized Crossover Trial. Am. J. Clin. Nutr..

[103] Kim Y.-Y., Jang K.-H., Lee E.-Y., Cho Y., Kang S.-A., Ha W.-K., Choue R. (2004). The Effect of Chicory Fructan Fiber on Calcium Absorption and Bone Metabolism in Korean Postmenopausal Women. Nutr. Sci..

[104] Abrams S.A., Griffin I.J., Hawthorne K.M., Liang L., Gunn S.K., Darlington G., Ellis K.J. (2005). A Combination of Prebiotic Short- and Long-Chain Inulin-Type Fructans Enhances Calcium Absorption and Bone Mineralization in Young Adolescents. Am. J. Clin. Nutr..

[105] McArdle P.D., Mellor D., Rilstone S., Taplin J. (2016). The Role of Carbohydrate in Diabetes Management. Pract. Diabetes.

[106] Jackuliak P., Payer J. (2014). Osteoporosis, Fractures, and Diabetes. Int. J. Endocrinol..

[107] Blahova J., Martiniakova M., Babikova M., Kovacova V., Mondockova V., Omelka R. (2021). Pharmaceutical Drugs and Natural Therapeutic Products for the Treatment of Type 2 Diabetes Mellitus. Pharmaceuticals.

[108] Martiniakova M., Blahova J., Kovacova V., Babikova M., Mondockova V., Kalafova A., Capcarova M., Omelka R. (2021). Bee Bread Can Alleviate Lipid Abnormalities and Impaired Bone Morphology in Obese Zucker Diabetic Rats. Molecules.

[109] Kadirvelu A., Gurtu S. (2013). Potential Benefits of Honey in Type 2 Diabetes Mellitus: A Review. Public Health.

[110] Kraemer K., Badham J., Christian P., Hyun Rah J. Sight and Life—Micronutrients, Macro Impact by Sight and Life. https://issuu.com/sight_and_life/docs/micronutriens_macro_impact.

[111] Cashman K.D. (2002). Calcium Intake, Calcium Bioavailability and Bone Health. Br. J. Nutr..

[112] Nieves J.W. (2005). Osteoporosis: The Role of Micronutrients. Am. J. Clin. Nutr..

[113] Winzenberg T., Shaw K., Fryer J., Jones G. (2006). Effects of Calcium Supplementation on Bone Density in Healthy Children: Meta-Analysis of Randomised Controlled Trials. BMJ.

[114] Cano A., Chedraui P., Goulis D.G., Lopes P., Mishra G., Mueck A., Senturk L.M., Simoncini T., Stevenson J.C., Stute P. (2018). Calcium in the Prevention of Postmenopausal Osteoporosis: EMAS Clinical Guide. Maturitas.

[115] Celotti F., Bignamini A. (1999). Dietary Calcium and Mineral/Vitamin Supplementation: A Controversial Problem. J. Int. Med. Res..

[116] Capozzi A., Scambia G., Lello S. (2020). Calcium, Vitamin D, Vitamin K2, and Magnesium Supplementation and Skeletal Health. Maturitas.

[117] Tai V., Leung W., Grey A., Reid I.R., Bolland M.J. (2015). Calcium Intake and Bone Mineral Density: Systematic Review and Meta-Analysis. BMJ.

[118] Price C.T., Langford J.R., Liporace F.A. (2012). Essential Nutrients for Bone Health and a Review of Their Availability in the Average North American Diet. Open Orthop. J..

[119] Sunyecz J.A. (2008). The Use of Calcium and Vitamin D in the Management of Osteoporosis. Ther. Clin. Risk Manag..

[120] Heaney R.P. (2006). Calcium Intake and Disease Prevention. Arq. Bras. Endocrinol. Metabol..

[121] Cashman K.D. (2007). Diet, Nutrition, and Bone Health. J. Nutr..

[122] Pastore S.M., Gomes P.C., Rostagno H.S., Albino L.F.T., Calderano A.A., Vellasco C.R., da Viana G.S., de Almeida R.L. (2012). Calcium Levels and Calcium: Available Phosphorus Ratios in Diets for White Egg Layers from 42 to 58 Weeks of Age. R. Bras. Zootec..

[123] Block G.D., Wood R.J., Allen L.H. (1980). A Comparison of the Effects of Feeding Sulfur Amino Acids and Protein on Urine Calcium in Man. Am. J. Clin. Nutr..

[124] Zemel M.B., Schuette S.A., Hegsted M., Linkswiler H.M. (1981). Role of the Sulfur-Containing Amino Acids in Protein-Induced Hypercalciuria in Men. J. Nutr..

[125] Straub D.A. (2007). Calcium Supplementation in Clinical Practice: A Review of Forms, Doses, and Indications. Nutr. Clin. Pract..

[126] Xu Y., Ye J., Zhou D., Su L. (2020). Research Progress on Applications of Calcium Derived from Marine Organisms. Sci. Rep..

[127] Świątkiewicz S., Arczewska-Włosek A., Krawczyk J., Puchała M., Józefiak D. (2015). Effects on Performance and Eggshell Quality of Particle Size of Calcium Sources in Laying Hens’ Diets with Different Ca Concentrations. Arch. Anim. Breed..

[128] Brennan O., Sweeney J., O’Meara B., Widaa A., Bonnier F.J., Byrne H.J., O’Gorman D.M. (2017). A Natural, Calcium-Rich Marine Multi-Mineral Complex Preserves Bone Structure, Composition and Strength in an Ovariectomised Rat Model of Osteoporosis. Calcif. Tissue Int..

[129] Omelka R., Martiniakova M., Svik K., Slovak L., Payer J., Oppenbergerova I., Kovacova V., Babikova M., Soltesova-Prnova M. (2021). The Effects of Eggshell Calcium (Biomin H^®^) and Its Combinations with Alfacalcidol (1α-Hydroxyvitamin D3) and Menaquinone-7 (Vitamin K2) on Ovariectomy-Induced Bone Loss in a Rat Model of Osteoporosis. J. Anim. Physiol. Anim. Nutr..

[130] Vorland C.J., Stremke E.R., Moorthi R.N., Hill Gallant K.M. (2017). Effects of Excessive Dietary Phosphorus Intake on Bone Health. Curr. Osteoporos. Rep..

[131] Ciosek Ż., Kot K., Kosik-Bogacka D., Łanocha-Arendarczyk N., Rotter I. (2021). The Effects of Calcium, Magnesium, Phosphorus, Fluoride, and Lead on Bone Tissue. Biomolecules.

[132] Butusov M., Jernelov A. (2013). Phosphorus. An Element That Could Have Been Called Lucifer.

[133] Kemi V.E., Kärkkäinen M.U.M., Lamberg-Allardt C.J.E. (2006). High Phosphorus Intakes Acutely and Negatively Affect Ca and Bone Metabolism in a Dose-Dependent Manner in Healthy Young Females. Br. J. Nutr..

[134] Kemi V.E., Rita H.J., Kärkkäinen M.U.M., Viljakainen H.T., Laaksonen M.M., Outila T.A., Lamberg-Allardt C.J.E. (2009). Habitual High Phosphorus Intakes and Foods with Phosphate Additives Negatively Affect Serum Parathyroid Hormone Concentration: A Cross-Sectional Study on Healthy Premenopausal Women. Public Health Nutr..

[135] Heaney R.P., Recker R.R., Watson P., Lappe J.M. (2010). Phosphate and Carbonate Salts of Calcium Support Robust Bone Building in Osteoporosis. Am. J. Clin. Nutr..

[136] Rafferty K., Heaney R.P. (2008). Nutrient Effects on the Calcium Economy: Emphasizing the Potassium Controversy. J. Nutr..

[137] Lee A.W., Cho S.S. (2015). Association between Phosphorus Intake and Bone Health in the NHANES Population. Nutr. J..

[138] Soetan K.O., Olaiya C.O., Oyewole O.E. (2010). The Importance of Mineral Elements for Humans, Domestic Animals and Plants—A Review. Afr. J. Food Sci..

[139] Koyama Y., Rittling S.R., Tsuji K., Hino K., Salincarnboriboon R., Yano T., Taketani Y., Nifuji A., Denhardt D.T., Noda M. (2006). Osteopontin Deficiency Suppresses High Phosphate Load-Induced Bone Loss via Specific Modulation of Osteoclasts. Endocrinology.

[140] Tucker K.L., Morita K., Qiao N., Hannan M.T., Cupples L.A., Kiel D.P. (2006). Colas, but Not Other Carbonated Beverages, Are Associated with Low Bone Mineral Density in Older Women: The Framingham Osteoporosis Study. Am. J. Clin. Nutr..

[141] Wyshak G. (2000). Teenaged Girls, Carbonated Beverage Consumption, and Bone Fractures. Arch. Pediatr. Adolesc. Med..

[142] Omelka R., Meliskova V., Conka J., Kovacova V., Sranko P., Celec P., Martiniakova M. No Effect of Long-Term Cola Intake on Quantitative Characteristics of Femoral Bone in Mice. Proceedings of the Osteoporosis International: WCO-IOF-ESCEO World Congress on Osteoporosis, Osteoarthritis and Musculoskeletal Diseases.

[143] Kristensen M., Jensen M., Kudsk J., Henriksen M., Mølgaard C. (2005). Short-Term Effects on Bone Turnover of Replacing Milk with Cola Beverages: A 10-Day Interventional Study in Young Men. Osteoporos. Int..

[144] Dennehy C., Tsourounis C. (2010). A Review of Select Vitamins and Minerals Used by Postmenopausal Women. Maturitas.

[145] Saris N.E., Mervaala E., Karppanen H., Khawaja J.A., Lewenstam A. (2000). Magnesium. An Update on Physiological, Clinical and Analytical Aspects. Clin. Chim. Acta.

[146] Leidi M., Dellera F., Mariotti M., Maier J.A.M. (2011). High Magnesium Inhibits Human Osteoblast Differentiation in Vitro. Magnes. Res..

[147] Boskey A.L., Rimnac C.M., Bansal M., Federman M., Lian J., Boyan B.D. (1992). Effect of Short-Term Hypomagnesemia on the Chemical and Mechanical Properties of Rat Bone. J. Orthop. Res..

[148] Belluci M.M., Giro G., Del Barrio R.A.L., Pereira R.M.R., Marcantonio E., Orrico S.R.P. (2011). Effects of Magnesium Intake Deficiency on Bone Metabolism and Bone Tissue around Osseointegrated Implants. Clin. Oral Implants Res..

[149] Orchard T.S., Larson J.C., Alghothani N., Bout-Tabaku S., Cauley J.A., Chen Z., LaCroix A.Z., Wactawski-Wende J., Jackson R.D. (2014). Magnesium Intake, Bone Mineral Density, and Fractures: Results from the Women’s Health Initiative Observational Study. Am. J. Clin. Nutr..

[150] Nieves J.W. (2013). Skeletal Effects of Nutrients and Nutraceuticals, beyond Calcium and Vitamin D. Osteoporos. Int..

[151] Houtkooper L.B., Ritenbaugh C., Aickin M., Lohman T.G., Going S.B., Weber J.L., Greaves K.A., Boyden T.W., Pamenter R.W., Hall M.C. (1995). Nutrients, Body Composition and Exercise Are Related to Change in Bone Mineral Density in Premenopausal Women. J. Nutr..

[152] Tucker K.L., Hannan M.T., Chen H., Cupples L.A., Wilson P.W., Kiel D.P. (1999). Potassium, Magnesium, and Fruit and Vegetable Intakes Are Associated with Greater Bone Mineral Density in Elderly Men and Women. Am. J. Clin. Nutr..

[153] Rude R.K., Singer F.R., Gruber H.E. (2009). Skeletal and Hormonal Effects of Magnesium Deficiency. J. Am. Coll. Nutr..

[154] Bancerz B., Duś-Żuchowska M., Cichy W., Matusiewicz H. (2013). Effect of Magnesium on Human Health. Gastroenterol. Rev..

[155] Dąbrowski M., Zioła-Frankowska A., Kubaszewski Ł., Rogala P., Frankowski M. (2018). Urban and Rural Area Differences in the Interaction between Oxidative Process Elements in Human Femoral Bone. Environ. Sci. Pollut. Res..

[156] Jurkiewicz A., Wiechuła D., Loska K. (2008). Original Article/Artykuł Oryginalny. J. Orthop. Trauma Surg. Rel. Res..

[157] Zioła-Frankowska A., Kubaszewski Ł., Dąbrowski M., Kowalski A., Rogala P., Strzyżewski W., Łabędź W., Uklejewski R., Novotny K., Kanicky V. (2015). The Content of the 14 Metals in Cancellous and Cortical Bone of the Hip Joint Affected by Osteoarthritis. BioMed Res. Int..

[158] Kuo H.W., Kuo S.M., Chou C.H., Lee T.C. (2000). Determination of 14 Elements in Taiwanese Bones. Sci. Total Environ..

[159] Ghishan F.K., Kiela P.R. (2017). Vitamins and Minerals in IBD. Gastroenterol. Clin. N. Am..

[160] Muñoz-Garach A., García-Fontana B., Muñoz-Torres M. (2020). Nutrients and Dietary Patterns Related to Osteoporosis. Nutrients.

[161] Ross A.C., Manson J.E., Abrams S.A., Aloia J.F., Brannon P.M., Clinton S.K., Durazo-Arvizu R.A., Gallagher J.C., Gallo R.L., Jones G. (2011). The 2011 Report on Dietary Reference Intakes for Calcium and Vitamin D from the Institute of Medicine: What Clinicians Need to Know. J. Clin. Endocrinol. Metab..

[162] Holick M.F., Binkley N.C., Bischoff-Ferrari H.A., Gordon C.M., Hanley D.A., Heaney R.P., Murad M.H., Weaver C.M. (2011). Endocrine Society Evaluation, Treatment, and Prevention of Vitamin D Deficiency: An Endocrine Society Clinical Practice Guideline. J. Clin. Endocrinol. Metab..

[163] Papadimitropoulos E., Wells G., Shea B., Gillespie W., Weaver B., Zytaruk N., Cranney A., Adachi J., Tugwell P., Josse R. (2002). Meta-Analyses of Therapies for Postmenopausal Osteoporosis. VIII: Meta-Analysis of the Efficacy of Vitamin D Treatment in Preventing Osteoporosis in Postmenopausal Women. Endocr. Rev..

[164] Bischoff-Ferrari H.A., Dawson-Hughes B., Willett W.C., Staehelin H.B., Bazemore M.G., Zee R.Y., Wong J.B. (2004). Effect of Vitamin D on Falls: A Meta-Analysis. JAMA.

[165] Warensjö E., Byberg L., Melhus H., Gedeborg R., Mallmin H., Wolk A., Michaëlsson K. (2011). Dietary Calcium Intake and Risk of Fracture and Osteoporosis: Prospective Longitudinal Cohort Study. BMJ.

[166] Boonen S., Lips P., Bouillon R., Bischoff-Ferrari H.A., Vanderschueren D., Haentjens P. (2007). Need for Additional Calcium to Reduce the Risk of Hip Fracture with Vitamin d Supplementation: Evidence from a Comparative Metaanalysis of Randomized Controlled Trials. J. Clin. Endocrinol. Metab..

[167] Chapuy M.C., Pamphile R., Paris E., Kempf C., Schlichting M., Arnaud S., Garnero P., Meunier P.J. (2002). Combined Calcium and Vitamin D3 Supplementation in Elderly Women: Confirmation of Reversal of Secondary Hyperparathyroidism and Hip Fracture Risk: The Decalyos II Study. Osteoporos. Int..

[168] Bolland M.J., Grey A., Avenell A. (2018). Effects of Vitamin D Supplementation on Musculoskeletal Health: A Systematic Review, Meta-Analysis, and Trial Sequential Analysis. Lancet Diabetes Endocrinol..

[169] Burt L.A., Billington E.O., Rose M.S., Raymond D.A., Hanley D.A., Boyd S.K. (2019). Effect of High-Dose Vitamin D Supplementation on Volumetric Bone Density and Bone Strength: A Randomized Clinical Trial. JAMA.

[170] Alwan A., Rizkallah M., Maalouf G., Matta J., Frenn F., Berro A.-J., Barakat A., Bachour F., Sebaaly A., Howayek M. (2018). Positive Correlations Between Free Vitamin D and Bone Variables in a Group of Young Lebanese Men. J. Clin. Densitom..

[171] Tang B.M.P., Eslick G.D., Nowson C., Smith C., Bensoussan A. (2007). Use of Calcium or Calcium in Combination with Vitamin D Supplementation to Prevent Fractures and Bone Loss in People Aged 50 Years and Older: A Meta-Analysis. Lancet.

[172] Cranney A., Horsley T., O’Donnell S., Weiler H., Puil L., Ooi D., Atkinson S., Ward L., Moher D., Hanley D. (2007). Effectiveness and Safety of Vitamin D in Relation to Bone Health. Evid. Rep. Technol. Assess. (Full Rep.).

[173] Chung M., Balk E.M., Brendel M., Ip S., Lau J., Lee J., Lichtenstein A., Patel K., Raman G., Tatsioni A. (2009). Vitamin D and Calcium: A Systematic Review of Health Outcomes. Evid. Rep. Technol. Assess. (Full Rep.).

[174] LeBoff M.S., Kohlmeier L., Hurwitz S., Franklin J., Wright J., Glowacki J. (1999). Occult Vitamin D Deficiency in Postmenopausal US Women with Acute Hip Fracture. JAMA.

[175] Tak Y.J., Lee S.Y. (2021). Anti-Obesity Drugs: Long-Term Efficacy and Safety: An Updated Review. World J. Men’s Health.

[176] Maurya V.K., Aggarwal M. (2017). Factors Influencing the Absorption of Vitamin D in GIT: An Overview. J. Food Sci. Technol..

[177] Pereira-Santos M., Costa P.R.F., Assis A.M.O., Santos C.A.S.T., Santos D.B. (2015). Obesity and Vitamin D Deficiency: A Systematic Review and Meta-Analysis. Obes. Rev..

[178] Need A.G., Morris H.A., Horowitz M., Nordin C. (1993). Effects of Skin Thickness, Age, Body Fat, and Sunlight on Serum 25-Hydroxyvitamin D. Am. J. Clin. Nutr..

[179] Migliaccio S., Di Nisio A., Mele C., Scappaticcio L., Savastano S., Colao A. (2019). Obesity Programs of nutrition, Education, Research and Assessment (OPERA) Group Obesity and Hypovitaminosis D: Causality or Casualty?. Int. J. Obes. Suppl..

[180] Sanders K.M., Stuart A.L., Williamson E.J., Simpson J.A., Kotowicz M.A., Young D., Nicholson G.C. (2010). Annual High-Dose Oral Vitamin D and Falls and Fractures in Older Women: A Randomized Controlled Trial. JAMA.

[181] Pludowski P., Holick M.F., Grant W.B., Konstantynowicz J., Mascarenhas M.R., Haq A., Povoroznyuk V., Balatska N., Barbosa A.P., Karonova T. (2018). Vitamin D Supplementation Guidelines. J. Steroid Biochem. Mol. Biol..

[182] Devaki S.J., Raveendran R.L. (2017). Vitamin C: Sources, Functions, Sensing and Analysis.

[183] Doseděl M., Jirkovský E., Macáková K., Krčmová L.K., Javorská L., Pourová J., Mercolini L., Remião F., Nováková L., Mladěnka P. (2021). Vitamin C-Sources, Physiological Role, Kinetics, Deficiency, Use, Toxicity, and Determination. Nutrients.

[184] Pehlivan F.E. (2017). Vitamin C: An Antioxidant Agent.

[185] Finck H., Hart A.R., Jennings A., Welch A.A. (2014). Is There a Role for Vitamin C in Preventing Osteoporosis and Fractures? A Review of the Potential Underlying Mechanisms and Current Epidemiological Evidence. Nutr. Res. Rev..

[186] Aghajanian P., Hall S., Wongworawat M.D., Mohan S. (2015). The Roles and Mechanisms of Actions of Vitamin C in Bone: New Developments. J. Bone Miner. Res..

[187] Brzezińska O., Łukasik Z., Makowska J., Walczak K. (2020). Role of Vitamin C in Osteoporosis Development and Treatment—A Literature Review. Nutrients.

[188] Fain O. (2005). Musculoskeletal Manifestations of Scurvy. Jt. Bone Spine.

[189] Simon J.A., Hudes E.S. (2001). Relation of Ascorbic Acid to Bone Mineral Density and Self-Reported Fractures among US Adults. Am. J. Epidemiol..

[190] Arslan A., Orkun S., Aydin G., Keles I., Tosun A., Arslan M., Caglayan O. (2011). Effects of Ovariectomy and Ascorbic Acid Supplement on Oxidative Stress Parameters and Bone Mineral Density in Rats. Libyan J. Med..

[191] Kim Y.A., Kim K.M., Lim S., Choi S.H., Moon J.H., Kim J.H., Kim S.W., Jang H.C., Shin C.S. (2015). Favorable Effect of Dietary Vitamin C on Bone Mineral Density in Postmenopausal Women (KNHANES IV, 2009): Discrepancies Regarding Skeletal Sites, Age, and Vitamin D Status. Osteoporos. Int..

[192] Ahmadieh H., Arabi A. (2011). Vitamins and Bone Health: Beyond Calcium and Vitamin D. Nutr. Rev..

[193] Chuin A., Labonté M., Tessier D., Khalil A., Bobeuf F., Doyon C.Y., Rieth N., Dionne I.J. (2009). Effect of Antioxidants Combined to Resistance Training on BMD in Elderly Women: A Pilot Study. Osteoporos. Int..

[194] Institute of Medicine (US) (2000). Panel on Dietary Antioxidants and Related Compounds In Dietary Reference Intakes for Vitamin C, Vitamin E, Selenium, and Carotenoids.

[195] Carr A.C., Frei B. (1999). Toward a New Recommended Dietary Allowance for Vitamin C Based on Antioxidant and Health Effects in Humans. Am. J. Clin. Nutr..

[196] Jacob R.A., Sotoudeh G. (2002). Vitamin C Function and Status in Chronic Disease. Nutr. Clin. Care.

[197] Carr A.C., Lykkesfeldt J. (2021). Discrepancies in Global Vitamin C Recommendations: A Review of RDA Criteria and Underlying Health Perspectives. Crit. Rev. Food Sci. Nutr..

[198] Taylor E.N., Stampfer M.J., Curhan G.C. (2004). Dietary Factors and the Risk of Incident Kidney Stones in Men: New Insights after 14 Years of Follow-Up. J. Am. Soc. Nephrol..

[199] Feskanich D., Weber P., Willett W.C., Rockett H., Booth S.L., Colditz G.A. (1999). Vitamin K Intake and Hip Fractures in Women: A Prospective Study. Am. J. Clin. Nutr..

[200] Lacombe J., Al Rifai O., Loter L., Moran T., Turcotte A.-F., Grenier-Larouche T., Tchernof A., Biertho L., Carpentier A.C., Prud’homme D. (2020). Measurement of Bioactive Osteocalcin in Humans Using a Novel Immunoassay Reveals Association with Glucose Metabolism and β-Cell Function. Am. J. Physiol. Endocrinol. Metab..

[201] Wei J., Karsenty G. (2015). An Overview of the Metabolic Functions of Osteocalcin. Rev. Endocr. Metab. Disord..

[202] Lin X., Brennan-Speranza T.C., Levinger I., Yeap B.B. (2018). Undercarboxylated Osteocalcin: Experimental and Human Evidence for a Role in Glucose Homeostasis and Muscle Regulation of Insulin Sensitivity. Nutrients.

[203] Beulens J.W.J., Bots M.L., Atsma F., Bartelink M.-L.E.L., Prokop M., Geleijnse J.M., Witteman J.C.M., Grobbee D.E., van der Schouw Y.T. (2009). High Dietary Menaquinone Intake Is Associated with Reduced Coronary Calcification. Atherosclerosis.

[204] Azuma K., Inoue S. (2019). Multiple Modes of Vitamin K Actions in Aging-Related Musculoskeletal Disorders. Int. J. Mol. Sci..

[205] Wu W.-J., Kim M.S., Ahn B.-Y. (2015). The Inhibitory Effect of Vitamin K on RANKL-Induced Osteoclast Differentiation and Bone Resorption. Food Funct..

[206] Azuma K., Ouchi Y., Inoue S. (2014). Vitamin K: Novel Molecular Mechanisms of Action and Its Roles in Osteoporosis. Geriatr. Gerontol. Int..

[207] Cockayne S., Adamson J., Lanham-New S., Shearer M.J., Gilbody S., Torgerson D.J. (2006). Vitamin K and the Prevention of Fractures: Systematic Review and Meta-Analysis of Randomized Controlled Trials. Arch. Intern. Med..

[208] Huang Z.-B., Wan S.-L., Lu Y.-J., Ning L., Liu C., Fan S.-W. (2015). Does Vitamin K2 Play a Role in the Prevention and Treatment of Osteoporosis for Postmenopausal Women: A Meta-Analysis of Randomized Controlled Trials. Osteoporos. Int..

[209] Su S., He N., Men P., Song C., Zhai S. (2019). The Efficacy and Safety of Menatetrenone in the Management of Osteoporosis: A Systematic Review and Meta-Analysis of Randomized Controlled Trials. Osteoporos. Int..

[210] Kanellakis S., Moschonis G., Tenta R., Schaafsma A., van den Heuvel E.G.H.M., Papaioannou N., Lyritis G., Manios Y. (2012). Changes in Parameters of Bone Metabolism in Postmenopausal Women Following a 12-Month Intervention Period Using Dairy Products Enriched with Calcium, Vitamin D, and Phylloquinone (Vitamin K(1)) or Menaquinone-7 (Vitamin K (2)): The Postmenopausal Health Study II. Calcif. Tissue Int..

[211] Šikuten I., Štambuk P., Andabaka Ž., Tomaz I., Marković Z., Stupić D., Maletić E., Kontić J.K., Preiner D. (2020). Grapevine as a Rich Source of Polyphenolic Compounds. Molecules.

[212] Pandey K.B., Rizvi S.I. (2009). Plant Polyphenols as Dietary Antioxidants in Human Health and Disease. Oxid. Med. Cell. Longev..

[213] Arts I.C.W., Hollman P.C.H. (2005). Polyphenols and Disease Risk in Epidemiologic Studies. Am. J. Clin. Nutr..

[214] Pojer E., Mattivi F., Johnson D., Stockley C.S. (2013). The Case for Anthocyanin Consumption to Promote Human Health: A Review. Compr. Rev. Food Sci. Food Saf..

[215] Minatel I.O., Borges C.V., Ferreira M.I., Gomez H.A.G., Lima C.-Y.O.C. (2017). Phenolic Compounds: Functional Properties, Impact of Processing and Bioavailability.

[216] Taleb-Contini S.H., Salvador M.J., Balanco J.M.F., Albuquerque S., de Oliveira D.C.R. (2004). Antiprotozoal Effect of Crude Extracts and Flavonoids Isolated from Chromolaena Hirsuta (Asteraceae). Phytother. Res..

[217] Wong S.K., Chin K.-Y., Ima-Nirwana S. (2019). The Osteoprotective Effects Of Kaempferol: The Evidence From In Vivo And In Vitro Studies. Drug Des. Dev. Ther..

[218] Sharma A.R., Nam J.-S. (2019). Kaempferol Stimulates WNT/β-Catenin Signaling Pathway to Induce Differentiation of Osteoblasts. J. Nutr. Biochem..

[219] Pang J.L., Ricupero D.A., Huang S., Fatma N., Singh D.P., Romero J.R., Chattopadhyay N. (2006). Differential Activity of Kaempferol and Quercetin in Attenuating Tumor Necrosis Factor Receptor Family Signaling in Bone Cells. Biochem. Pharmacol..

[220] Hirata M., Matsumoto C., Takita M., Miyaura C., Inada M. (2009). Naringin Suppresses Osteoclast Formation and Enhances Bone Mass in Mice. J. Health Sci..

[221] Wang Q.-L., Huo X.-C., Wang J.-H., Wang D.-P., Zhu Q.-L., Liu B., Xu L.-L. (2017). Rutin Prevents the Ovariectomy-Induced Osteoporosis in Rats. Eur. Rev. Med. Pharmacol. Sci..

[222] Gera S., Pooladanda V., Godugu C., Swamy Challa V., Wankar J., Dodoala S., Sampathi S. (2020). Rutin Nanosuspension for Potential Management of Osteoporosis: Effect of Particle Size Reduction on Oral Bioavailability, in Vitro and in Vivo Activity. Pharm. Dev. Technol..

[223] Kim T.-H., Jung J.W., Ha B.B.G., Hong J.M., Park E.K., Kim H.-J., Kim S.-Y. (2011). The Effects of Luteolin on Osteoclast Differentiation, Function in Vitro and Ovariectomy-Induced Bone Loss. J. Nutr. Biochem..

[224] Jing Z., Wang C., Yang Q., Wei X., Jin Y., Meng Q., Liu Q., Liu Z., Ma X., Liu K. (2019). Luteolin Attenuates Glucocorticoid-Induced Osteoporosis by Regulating ERK/Lrp-5/GSK-3β Signaling Pathway in Vivo and in Vitro. J. Cell. Physiol..

[225] Vakili S., Zal F., Mostafavi-pour Z., Savardashtaki A., Koohpeyma F. (2021). Quercetin and Vitamin E Alleviate Ovariectomy-Induced Osteoporosis by Modulating Autophagy and Apoptosis in Rat Bone Cells. J. Cell. Physiol..

[226] Li L., Zeng Z., Cai G. (2011). Comparison of Neoeriocitrin and Naringin on Proliferation and Osteogenic Differentiation in MC3T3-E1. Phytomedicine.

[227] Niu Y., Yang Y., Xiao X., Sun Y., Zhou Y., Zhang Y., Dong D., Li C., Wu X., Li Y. (2020). Quercetin Prevents Bone Loss in Hindlimb Suspension Mice via Stanniocalcin 1-Mediated Inhibition of Osteoclastogenesis. Acta Pharmacol. Sin..

[228] Nishimuro H., Ohnishi H., Sato M., Ohnishi-Kameyama M., Matsunaga I., Naito S., Ippoushi K., Oike H., Nagata T., Akasaka H. (2015). Estimated Daily Intake and Seasonal Food Sources of Quercetin in Japan. Nutrients.

[229] Tsuji M., Yamamoto H., Sato T., Mizuha Y., Kawai Y., Taketani Y., Kato S., Terao J., Inakuma T., Takeda E. (2009). Dietary Quercetin Inhibits Bone Loss without Effect on the Uterus in Ovariectomized Mice. J. Bone Miner. Metab..

[230] Yuan Z., Min J., Zhao Y., Cheng Q., Wang K., Lin S., Luo J., Liu H. (2018). Quercetin Rescued TNF-Alpha-Induced Impairments in Bone Marrow-Derived Mesenchymal Stem Cell Osteogenesis and Improved Osteoporosis in Rats. Am. J. Transl. Res..

[231] Abd El-Fattah A.I., Fathy M.M., Ali Z.Y., El-Garawany A.E.-R.A., Mohamed E.K. (2017). Enhanced Therapeutic Benefit of Quercetin-Loaded Phytosome Nanoparticles in Ovariectomized Rats. Chem. Biol. Interact..

[232] Ge Y., Feng K., Liu X., Zhu Z., Chen H., Chang Y., Sun Z., Wang H., Zhang J., Yu D. (2020). Quercetin Inhibits Macrophage Polarization through the P-38α/β Signalling Pathway and Regulates OPG/RANKL Balance in a Mouse Skull Model. J. Cell. Mol. Med..

[233] Yamaguchi M., Weitzmann M.N. (2011). Quercetin, a Potent Suppressor of NF-ΚB and Smad Activation in Osteoblasts. Int. J. Mol. Med..

[234] Woo J.-T., Nakagawa H., Notoya M., Yonezawa T., Udagawa N., Lee I.-S., Ohnishi M., Hagiwara H., Nagai K. (2004). Quercetin Suppresses Bone Resorption by Inhibiting the Differentiation and Activation of Osteoclasts. Biol. Pharm. Bull..

[235] Tripathi G., Raja N., Yun H.S. (2015). Effect of Direct Loading of Phytoestrogens into the Calcium Phosphate Scaffold on Osteoporotic Bone Tissue Regeneration. J. Mater. Chem. B.

[236] Kim D.-S., Takai H., Arai M., Araki S., Mezawa M., Kawai Y., Murota K., Terao J., Ogata Y. (2007). Effects of Quercetin and Quercetin 3-Glucuronide on the Expression of Bone Sialoprotein Gene. J. Cell. Biochem..

[237] Siddiqui J.A., Swarnkar G., Sharan K., Chakravarti B., Gautam A.K., Rawat P., Kumar M., Gupta V., Manickavasagam L., Dwivedi A.K. (2011). A Naturally Occurring Rare Analog of Quercetin Promotes Peak Bone Mass Achievement and Exerts Anabolic Effect on Osteoporotic Bone. Osteoporos. Int..

[238] Prouillet C., Mazière J.-C., Mazière C., Wattel A., Brazier M., Kamel S. (2004). Stimulatory Effect of Naturally Occurring Flavonols Quercetin and Kaempferol on Alkaline Phosphatase Activity in MG-63 Human Osteoblasts through ERK and Estrogen Receptor Pathway. Biochem. Pharmacol..

[239] Ross J.A., Kasum C.M. (2002). Dietary Flavonoids: Bioavailability, Metabolic Effects, and Safety. Annu. Rev. Nutr..

[240] Wang T., Liu Q., Tjhioe W., Zhao J., Lu A., Zhang G., Tan R.X., Zhou M., Xu J., Feng H.T. (2017). Therapeutic Potential and Outlook of Alternative Medicine for Osteoporosis. Curr. Drug Targets.

[241] Wang N., Wang L., Yang J., Wang Z., Cheng L. (2021). Quercetin Promotes Osteogenic Differentiation and Antioxidant Responses of Mouse Bone Mesenchymal Stem Cells through Activation of the AMPK/SIRT1 Signaling Pathway. Phytother. Res..

[242] Sharma S., Ali A., Ali J., Sahni J.K., Baboota S. (2013). Rutin: Therapeutic Potential and Recent Advances in Drug Delivery. Expert Opin. Investig. Drugs.

[243] Lee H.-H., Jang J.-W., Lee J.-K., Park C.-K. (2020). Rutin Improves Bone Histomorphometric Values by Reduction of Osteoclastic Activity in Osteoporosis Mouse Model Induced by Bilateral Ovariectomy. J. Korean Neurosurg. Soc..

[244] Kaptoge S., Beck T.J., Reeve J., Stone K.L., Hillier T.A., Cauley J.A., Cummings S.R. (2008). Prediction of Incident Hip Fracture Risk by Femur Geometry Variables Measured by Hip Structural Analysis in the Study of Osteoporotic Fractures. J. Bone Miner. Res..

[245] Kyung T.-W., Lee J.-E., Shin H.-H., Choi H.-S. (2008). Rutin Inhibits Osteoclast Formation by Decreasing Reactive Oxygen Species and TNF-α by Inhibiting Activation of NF-ΚB. Exp. Mol. Med..

[246] Middleton E.T., Steel S.A., Aye M., Doherty S.M. (2010). The Effect of Prior Bisphosphonate Therapy on the Subsequent BMD and Bone Turnover Response to Strontium Ranelate. J. Bone Miner. Res..

[247] Horcajada-Molteni M.N., Crespy V., Coxam V., Davicco M.J., Rémésy C., Barlet J.P. (2000). Rutin Inhibits Ovariectomy-Induced Osteopenia in Rats. J. Bone Miner. Res..

[248] Xiao Y., Wei R., Yuan Z., Lan X., Kuang J., Hu D., Song Y., Luo J. (2019). Rutin Suppresses FNDC1 Expression in Bone Marrow Mesenchymal Stem Cells to Inhibit Postmenopausal Osteoporosis. Am. J. Transl. Res..

[249] Kotanidou A., Xagorari A., Bagli E., Kitsanta P., Fotsis T., Papapetropoulos A., Roussos C. (2002). Luteolin Reduces Lipopolysaccharide-Induced Lethal Toxicity and Expression of Proinflammatory Molecules in Mice. Am. J. Respir. Crit. Care Med..

[250] Fatokun A.A., Tome M., Smith R.A., Darlington L.G., Stone T.W. (2015). Protection by the Flavonoids Quercetin and Luteolin against Peroxide- or Menadione-Induced Oxidative Stress in MC3T3-E1 Osteoblast Cells. Nat. Prod. Res..

[251] Xagorari A., Papapetropoulos A., Mauromatis A., Economou M., Fotsis T., Roussos C. (2001). Luteolin Inhibits an Endotoxin-Stimulated Phosphorylation Cascade and Proinflammatory Cytokine Production in Macrophages. J. Pharmacol. Exp. Ther..

[252] Choi E.-M. (2007). Modulatory Effects of Luteolin on Osteoblastic Function and Inflammatory Mediators in Osteoblastic MC3T3-E1 Cells. Cell Biol. Int..

[253] Kim S.-Y., Jung J.-W., Kim T.-H., Hong J.M., Kim H.-J., Park E.K. Effect of Luteolin on Bone Resorption, Bone Loss and Microarchitecture in Ovariectomized Mice. Proceedings of the 55th Annual Meeting of the Orthopaedic Research Society.

[254] Calderón-Montaño J.M., Burgos-Morón E., Pérez-Guerrero C., López-Lázaro M. (2011). A Review on the Dietary Flavonoid Kaempferol. Mini Rev. Med. Chem..

[255] Imran M., Rauf A., Shah Z.A., Saeed F., Imran A., Arshad M.U., Ahmad B., Bawazeer S., Atif M., Peters D.G. (2019). Chemo-Preventive and Therapeutic Effect of the Dietary Flavonoid Kaempferol: A Comprehensive Review. Phytother. Res..

[256] Trivedi R., Kumar S., Kumar A., Siddiqui J.A., Swarnkar G., Gupta V., Kendurker A., Dwivedi A.K., Romero J.R., Chattopadhyay N. (2008). Kaempferol Has Osteogenic Effect in Ovariectomized Adult Sprague-Dawley Rats. Mol. Cell. Endocrinol..

[257] Nowak B., Matuszewska A., Nikodem A., Filipiak J., Landwójtowicz M., Sadanowicz E., Jędrzejuk D., Rzeszutko M., Zduniak K., Piasecki T. (2017). Oral Administration of Kaempferol Inhibits Bone Loss in Rat Model of Ovariectomy-Induced Osteopenia. Pharmacol. Rep..

[258] Liu H., Yi X., Tu S., Cheng C., Luo J. (2021). Kaempferol Promotes BMSC Osteogenic Differentiation and Improves Osteoporosis by Downregulating MiR-10a-3p and Upregulating CXCL12. Mol. Cell. Endocrinol..

[259] Kim C.-J., Shin S.-H., Kim B.-J., Kim C.-H., Kim J.-H., Kang H.-M., Park B.-S., Kim I.-R. (2018). The Effects of Kaempferol-Inhibited Autophagy on Osteoclast Formation. Int. J. Mol. Sci..

[260] Jia M., Nie Y., Cao D.-P., Xue Y.-Y., Wang J.-S., Zhao L., Rahman K., Zhang Q.-Y., Qin L.-P. (2012). Potential Antiosteoporotic Agents from Plants: A Comprehensive Review. Evid. Based Complement. Altern. Med..

[261] Tang X., Zhu X., Liu S., Nicholson R.C., Ni X. (2008). Phytoestrogens Induce Differential Estrogen Receptor β-Mediated Responses in Transfected MG-63 Cells. Endocrine.

[262] Yang L., Chen Q., Wang F., Zhang G. (2011). Antiosteoporotic Compounds from Seeds of Cuscuta Chinensis. J. Ethnopharmacol..

[263] Alam M.A., Subhan N., Rahman M.M., Uddin S.J., Reza H.M., Sarker S.D. (2014). Effect of Citrus Flavonoids, Naringin and Naringenin, on Metabolic Syndrome and Their Mechanisms of Action. Adv. Nutr..

[264] Yu K.E., Alder K.D., Morris M.T., Munger A.M., Lee I., Cahill S.V., Kwon H.-K., Back J., Lee F.Y. (2020). Re-Appraising the Potential of Naringin for Natural, Novel Orthopedic Biotherapies. Ther. Adv. Musculoskelet..

[265] Wang D.-M., Yang Y.-J., Zhang L., Zhang X., Guan F.-F., Zhang L.-F. (2013). Naringin Enhances CaMKII Activity and Improves Long-Term Memory in a Mouse Model of Alzheimer’s Disease. Int. J. Mol. Sci..

[266] Pang W.-Y., Wang X.-L., Mok S.-K., Lai W.-P., Chow H.-K., Leung P.-C., Yao X.-S., Wong M.-S. (2010). Naringin Improves Bone Properties in Ovariectomized Mice and Exerts Oestrogen-like Activities in Rat Osteoblast-like (UMR-106) Cells. Br. J. Pharmacol..

[267] Wang D., Ma W., Wang F., Dong J., Wang D., Sun B., Wang B. (2015). Stimulation of Wnt/β-Catenin Signaling to Improve Bone Development by Naring.gin via Interacting with AMPK and Akt. Cell. Physiol. Biochem..

[268] Li N., Jiang Y., Wooley P.H., Xu Z., Yang S.-Y. (2013). Naringin Promotes Osteoblast Differentiation and Effectively Reverses Ovariectomy-Associated Osteoporosis. J. Orthop. Sci..

[269] Zhu Z., Xie W., Li Y., Zhu Z., Zhang W. (2021). Effect of Naringin Treatment on Postmenopausal Osteoporosis in Ovariectomized Rats: A Meta-Analysis and Systematic Review. Evid. Based Complement. Altern. Med..

[270] Wu J.-B., Fong Y.-C., Tsai H.-Y., Chen Y.-F., Tsuzuki M., Tang C.-H. (2008). Naringin-Induced Bone Morphogenetic Protein-2 Expression via PI3K, Akt, c-Fos/c-Jun and AP-1 Pathway in Osteoblasts. Eur. J. Pharmacol..

[271] Wong R.W.K., Rabie A.B.M. (2006). Effect of Naringin on Bone Cells. J. Orthop. Res..

